# Pre-Event Self-Efficacy and Sports Performance: A Systematic Review with Meta-Analysis

**DOI:** 10.3390/sports11110222

**Published:** 2023-11-12

**Authors:** Marc Lochbaum, Cassandra Sisneros, Sydney Cooper, Peter C. Terry

**Affiliations:** 1 Department of Kinesiology and Sport Management, Texas Tech University, Lubbock, TX 79409, USA; cassandra.sisneros@ttu.edu; 2 Research Institute, Education Academy, Vytautas Magnus University, 44248 Kaunas, Lithuania; 3 Honors College, Texas Tech University, Lubbock, TX 79409, USA; sydneyco@ttu.edu; 4 School of Psychology and Wellbeing, University of Southern Queensland, Toowoomba, QLD 4350, Australia; peter.terry@unisq.edu.au; 5 Centre for Health Research, University of Southern Queensland, Toowoomba, QLD 4350, Australia

**Keywords:** confidence, competitive sports, athletes, quantitative review

## Abstract

The relationship between self-efficacy and performance exclusively within the sports environment is yet to be quantified. Hence, we meta-analysed this relationship by following the PRISMA guidelines. Two previous meta-analyses, five relevant databases, and Google Scholar were searched. Forty-four articles published between 1983 and 2021 met the inclusion criteria, with 55 independent samples. Comprehensive meta-analysis software version 4 was used for all meta-analytic calculations using a random-effects model to calculate the mean effect size, and a mixed-effects model was used for moderation analyses. The mean pre-event self-efficacy and performance effect size was *r* = 0.31 (95% CI 0.22, 0.40). For moderation analyses, notable mean differences (*p* values ≥ 0.08) resulted for concordance [concordant (*r* = 0.37), nonconcordant (*r* = 0.22)], sports skill [closed (*r* = 0.37), open (*r* = 0.23)], and athlete level [elite (*r* = 0.40), sub-elite (*r* = 0.28)]. The true effect prediction interval ranged from negative (i.e., self-efficacy impairing performance) to positive (self-efficacy improving performance) for all moderator variables except self-referenced vs. other-referenced performance. In conclusion, the relationship between pre-event self-efficacy and performance is positive and moderate in magnitude, although with prediction intervals ranging from debilitating to facilitating performance. Researchers and practitioners should note that high athlete-rated self-efficacy might not always improve impending competitive sports performance.

## 1. Introduction

Ever since Bandura [1] produced his seminal publication, titled Self-efficacy: Toward a Unifying Theory of Behavioral Change, self-efficacy has been a prominent motivation construct in achievement domains. The construct of self-efficacy refers to an individual’s belief in their own ability to perform a specific task or achieve a specific goal, compared to the self-confidence construct, which is a broader belief in one’s overall abilities and personal qualities [1]. Across many decades of research, findings suggest that self-efficacy is a key factor in sports performance [2]. Athletes with high self-efficacy are more likely to set challenging goals, persist in the face of setbacks, and perform better under pressure [2]. In contrast, athletes with low self-efficacy are more likely to set easier goals, avoid difficult tasks, and have lower levels of motivation [2].

According to Bandura [1], self-efficacy is formed based on four main sources of information: mastery experiences (successful performance in the past), which is the most authentic and influential source of self-efficacy; vicarious experiences (observing others succeed); social persuasion (verbal encouragement or feedback from others); and control of physiological and emotional states (such as anxiety or stress). Maddux [3] subsequently introduced a fifth source of self-efficacy, referred to as imaginal experiences, or visualization. Researchers have studied the sources and effects of self-efficacy extensively in both sports [2,4,5] and physical activity [6,7] domains since the 1980s.

In the sports and physical activity domains, sufficient published studies were accumulated by the late 1990s to warrant a meta-analysis being conducted by Moritz and colleagues [8]. Their results showed a mean correlation between self-efficacy and sports performance of 0.38, based on 45 published studies in sports plus several other physical activity domains. Other meta-analyses before and after Moritz et al. [8] reported an identical [9] or near-identical [10] relationship between self-efficacy and work performance and self-efficacy and academic achievement, respectively. Moritz and her colleagues reported concordance between self-efficacy and performance measures as the strongest moderator of the efficacy–performance relationship, which accounted for 12.6% of the variance. Moritz et al. [8] also reported that differences in the self-efficacy measure (task-specific, domain-specific, single-item), performance measure (subjective, objective, self-report), nature of the task (novel, familiar), and time of assessment in relation to performance (before, after) were all significant moderators of the effect of self-efficacy on performance.

The meta-analysis by Moritz et al. [8] was published 23 years ago at the time of writing this manuscript, and many studies investigating the efficacy–performance relationship have been published during the intervening period. Moreover, in a recent self-confidence and sports performance review, Jekauc and colleagues [11] included just four studies that assessed self-efficacy. Taken together, a systematic review with meta-analysis specific to pre-event self-efficacy assessment and competitive sports is still absent from the literature, given that Moritz and colleagues included several non-sports samples and also included post-performance efficacy and Jekauc and colleagues included very few self-efficacy and sports performance samples. Such an omission represents a significant gap in the sports psychology and performance meta-analysis literature [12]. Hence, we extended the sports psychology and performance literature by conducting a systematic review using a meta-analysis of the pre-event self-efficacy and competitive sports literature. We delimited our review to studies with explicit statements, indicating that self-efficacy was assessed prior to sports competitions, either actual or contrived. We excluded studies of related constructs, such as state self-confidence [13], self-efficacy assessed post-performance, and self-efficacy assessed in relation to physical activities (e.g., vertical jump) or sports-related tests (e.g., agility run).

### 1.1. Moderating Variables

In addition to quantifying the overall pre-event efficacy and sports performance relationship, we examined several characteristics of the sports and the participants that have been proposed to moderate the relationship between pre-event psychological states and sports performance outcome [14]. In terms of the type of sport, the pre-event self-efficacy and performance relationship may be stronger in sports like diving [15], which are performed in a predictable, unchanging environment, referred to as closed-skill sports, compared to sports like soccer [16], which are performed in a dynamic, rapidly changing environment, referred to as open-skilled sports, because the direct influence of opponents on performance is absent. Second, the potential for self-efficacy to vary during the performance is greater in long-duration sports than in short-duration sports. Therefore, the pre-event self-efficacy and performance relationship may be stronger in short-duration sports such as sprints [17] than in long-duration sports such as the triathlon [18] because performance outcomes in short-duration sports occur in closer temporal proximity to the assessment of self-efficacy. Related to the type of sport, although not hypothesized in the literature, we coded each sports competition as actual or contrived to assess whether the pre-event self-efficacy and performance relationship differed between the two. From an applied practitioner perspective, it would be important for the evidence to indicate that the pre-event self-efficacy and performance relationship is at least as strong in actual sporting events as it is in contrived competitions.

Regarding the performance measure, it has been proposed [14] that the relationship between psychological variables, including self-efficacy, and sports performance may be stronger if the performance is self-referenced or relative to previous performances by the same athlete, such as running a personal best time, rather than other-referenced or relative to the performance of other athletes, such as the finish position. Similarly, the relationship may be stronger if the performance measure is subjective (e.g., coach-rated or self-rated) rather than objective (e.g., finish position or finish time). Although objective measures are more precise indicators of performance, subjective ratings are proposed to be more sensitive to individual fluctuations in performance [14]. In previous meta-analyses, subjective performance measures have been shown to be more closely related to some pre-competition psychological states, such as moods, than objective performance measures [19,20], although this finding was not supported in the self-confidence literature [13].

In terms of the characteristics of the athlete, Terry [14] proposed that the relative ability and fitness of those competing are influential. For example, Olympic champion sprinters will beat club sprinters simply due to their superior ability. In such scenarios, psychological factors play no, or almost no, role in determining the outcome. However, among athletes who are similar in terms of ability and fitness such as the eight finalists of an Olympic 100 m race, psychological factors play a more decisive role in determining winners and losers. It is proposed, therefore, that psychological factors, such as self-efficacy, are more closely related to performance at the elite level, where athletes tend to have relatively similar levels of ability and fitness, than at the sub-elite level, where the ability and fitness of participants may vary widely. However, this proposition was not supported in a recent meta-analysis that focused on self-confidence in sports [13], in which athlete level did not moderate the relationship with performance. Finally, the self-efficacy and performance relationship may be moderated by the sex of the athlete as reported for the self-confidence and sports performance relationship [13].

### 1.2. Aims of the Study

The main aim of the meta-analysis was to quantify the overall strength and robustness of the relationship (i.e., the mean standardized effect and sensitivity analysis) between pre-event self-efficacy and subsequent sports performance. Our secondary aims, insofar as the methodological details provided in the primary studies facilitated analysis, were to explore the moderating influence of the characteristics of the sports event (duration; skill type; actual, contrived), the participants involved (elite, sub-elite; male, female), and the measures used to assess sports performance (objective, subjective; other-referenced, self-referenced), and self-efficacy (concordant, non-concordant).

## 2. Materials and Methods

This systematic review with meta-analysis followed the Preferred Reporting Items for Systematic Reviews and Meta-Analysis (PRISMA) guidelines [21] (see Supplement Table S1 for specifics). We performed the meta-analysis using Borenstein, Hedges, Higgins, and Rothstein’s [22] Comprehensive Meta-Analyses (CMA) Version 4 program along with the provided interpretation suggestions [23].

### 2.1. Eligibility Criteria and Selection Process

The included studies met the following criteria: (a) participants engaged in a competitive sports event or contest; (b) a reported self-efficacy measure; (c) a reported measure of sports performance; (d) a statement that self-efficacy was assessed prior to the sports competition; (e) sufficient data provided for effect size calculation; and (f) original data published in a peer-reviewed scholarly journal. We excluded studies conducted in a non-sports setting such as a physical education class and excluded studies that used physical performance measures such as vertical jump height rather than a measure of sports performance. We also excluded studies that assessed self-confidence rather than self-efficacy, as indicated by the questionnaire described in the methods section of the studies. We did not inquire about missing data or clarifications. If an article was not in English, we used Google Translate https://translate.google.com/ (last accessed 6 June 2022) to assist in identifying pertinent information.

### 2.2. Information Sources, Search Strategy, and Search Protocol

**Figure 1:**
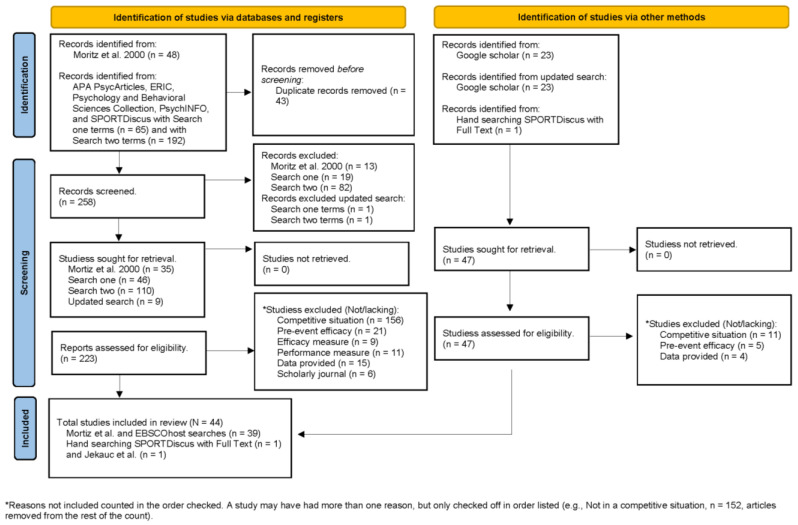
PRISMA flow chart for the identification of the included studies [15,16,17,18,24,25,26,27,28,29,30,31,32,33,34,35,36,37,38,39,40,41,42,43,44,45,46,47,48,49,50,51,52,53,54,55,56,57,58,59,60,61,62,63].

Information sources (see Figure 1) included references from the Moritz et al. [8] and Jekauc et al. [11] meta-analyses, databases found within EBSCOhost (first search ended 15 September 2022 and second search covering 2022–2023 ended 1 September 2023), and Google Scholar (first search ended 26 September 2022 and second search covering 2022–2023 ended 1 September 2023). The specific databases within EBSCOhost were APA PsycArticles, ERIC, Psychology and Behavioral Sciences Collection, PsychINFO, and SPORTDiscus. Supplement Table S2 contains the records of all articles from the searches. We performed all searches year by year starting from 1977 corresponding to Bandura’s [1] publication in which the term “self-efficacy” was first used, which provided the impetus for future self-efficacy research. The following represents our searches:

1. Examined all of Moritz et al.’s [8] studies.
2. Began the EBSCOhost search.
3. Selected individual databases (e.g., PsychINFO).
4. Selected EBSCOhost advanced search.
5. Typed in the following search terms using the Boolean operator AND: self-efficacy AND sport performance AND competitive.
6. Limited EBSCOhost to scholarly peer-reviewed journals.
7. Selected page options for 50 records per page.
8. Limited search time for a specific year (e.g., 1996).
9. Repeated until all years of the search were completed.
10. Began another search, substituting athlete for competitive with the Boolean operator AND.
11. Opened Google Scholar at https://scholar.google.com (last accessed 26 September 2022)
12. Typed self-efficacy and sport performance.
13. Limited year to one (e.g., 2010).
14. Searched the first three pages of the results.
15. Repeated all searches covering 2022–2023 as time passed, completing our manuscript.
16. Examined Jekauc et al.’s [11] included studies.
17. Hand searched SPORTDiscus.

### 2.3. Data Collection and Items Retrieved

Based on past co-authored works [13,20,64], the first and fourth authors developed a data collection worksheet. Then, in pairs (e.g., first and second author, first and fourth author), we extracted the following information: participants’ age, total number, percent male, country, sport, participant level description (i.e., Olympic athlete, collegiate athlete); self-efficacy measure description; sports performance characteristics (e.g., time and placement, team statistics); and data available (i.e., mean scores or correlation).

### 2.4. Study Quality and Risk of Bias Assessments

We pre-planned to examine whether study quality (i.e., lower quality studies biased the results with a higher effect size) impacted the pre-event self-efficacy and performance relationship. Table 1 contains the study quality questions and rating explanations. The categories and questions stemmed from Hoy and colleagues’ [65] assessment tool and from Lochbaum and colleagues’ [13] confidence and sports performance meta-analysis. The second author rated all studies with a discussion of all ratings with the first author.

For the risk of bias across studies, the classic fail-safe *n*, Orwin’s fail-safe *n*, the funnel plot, and Duval and Tweedie’s [66] trim and fill results were examined. The classic fail-safe *n* statistic represents the number of null samples required to change a significant value into a non-significant value [67]. We specified the one-tailed test when we conducted the classic fail-safe *n* analysis. Orwin’s fail-safe *n* [68] is the number of potential missed studies that, when added to the actual data, would move the new correlation past a chosen threshold. We chose zero as our missed study value and 0.10 as our threshold, as this value is the lower end of a correlation with small meaningfulness. Hence, the greater the value for both fail-safe *n* calculations, the greater the confidence that the result is safe from publication bias. To determine whether the entered studies dispersed in a similar manner on either side of the overall effect, we examined funnel plots [69]. Full plot symmetry indicates that the retrieved studies captured the essence of all studies. Lastly, we examined Duval and Tweedie’s [66] trim and fill analysis, which is used to adjust for potential missing studies. If required, data points filled to the right increase the effect size value, whereas those filled to the left lower the effect size value.

### 2.5. Summary Statistics, Planned Analyses, and Certainty Assessment

The random-effects model was the summary statistic, given our studies are best thought of as a random sampling of studies in the literature [22,23] using the correlation coefficient (*r*) as the primary effect size parameter. Cohen’s [70] guidelines for correlation values of 0.10–0.29 as small, 0.30–0.49 as medium, and 0.50 or greater as large, defined meaningfulness. For the main pre-event self-efficacy and performance analysis, we reported the number of samples, total number of participants, mean effect size, 95% confidence and prediction intervals, two heterogeneity statistics, Tau-squared (τ^2^) and I-squared (*I^2^*), and publication bias statistics. We used a mixed-effects analysis for our moderator analyses. For the moderator analyses, we reported the number of samples, the mean effect size, 95% confidence and prediction intervals, and the Q total between statistics with the associated *p*-value. The Q total indicates the level of difference between different moderator levels. Moderator analyses with small and unequal samples are unpowered to assess for differences [71]. Though we set statistical significance at the traditional *p* < 0.05, our hypotheses with our research questions such as Terry’s [14] propositions predict trends rather than statistical differences. We used a random-effects meta-regression model to test the impact of the study quality scores and the percentage of male participants on the pre-event self-efficacy and performance relationship.

To assess the robustness of our results, we conducted two sensitivity analyses provided in the CMA program in addition to the classic fail-safe *n* and Orwin’s fail-safe *n*, both of which provide statistics indicating robustness. First, we ran the CMA remove-one analysis to gauge each study’s impact. The remove-one analysis runs the data with all studies except the first, and then all studies except the second, and so on with the resulting plot depicting the impact of each study. Next, we ran the CMA cumulative analysis program by study publication year. The cumulative analysis run by year allowed us to determine the consistency of the self-efficacy to sports performance relationship over time using the forest plots. Lastly, we examined our results (e.g., 95% confidence and prediction intervals, risk of bias assessments, and differences between moderator groups) with the aim of assessing certainty related to our research question hypotheses.

## 3. Results

### 3.1. Study Selection, Characteristics, and Quality

Table 2 contains details of the 44 included studies, of which some used more than one sample, resulting in 55 independent samples. As reported in Figure 1, the most common reason for excluding studies was being conducted in non-competitive sports situations. The study publication years spanned from 1983 to 2021, with multiple studies from each decade; 1980s (*n* = 4), 1990s (*n* = 12), 2000s (*n* = 11), 2010s (*n* = 13), and 2020s (*n* = 5). In total, the studies included 5373 participants ranging from 6 to 416 participants in a sample from 12 countries in the following continents: Australasia (Australia), Europe (France, Greece, Italy, Poland, Spain, UK), Asia (Islamic Republic of Iran, Israel, and Japan), and North America (Canada, USA). Participants included children, adolescents, and adults. Of samples reporting male and female composition, 35 were at least greater than 50% male participants. Studies reported on both individual sports athletes (e.g., golf, running, and tennis) and team sports athletes (e.g., basketball, soccer, and ice hockey).

**Figure 2:**
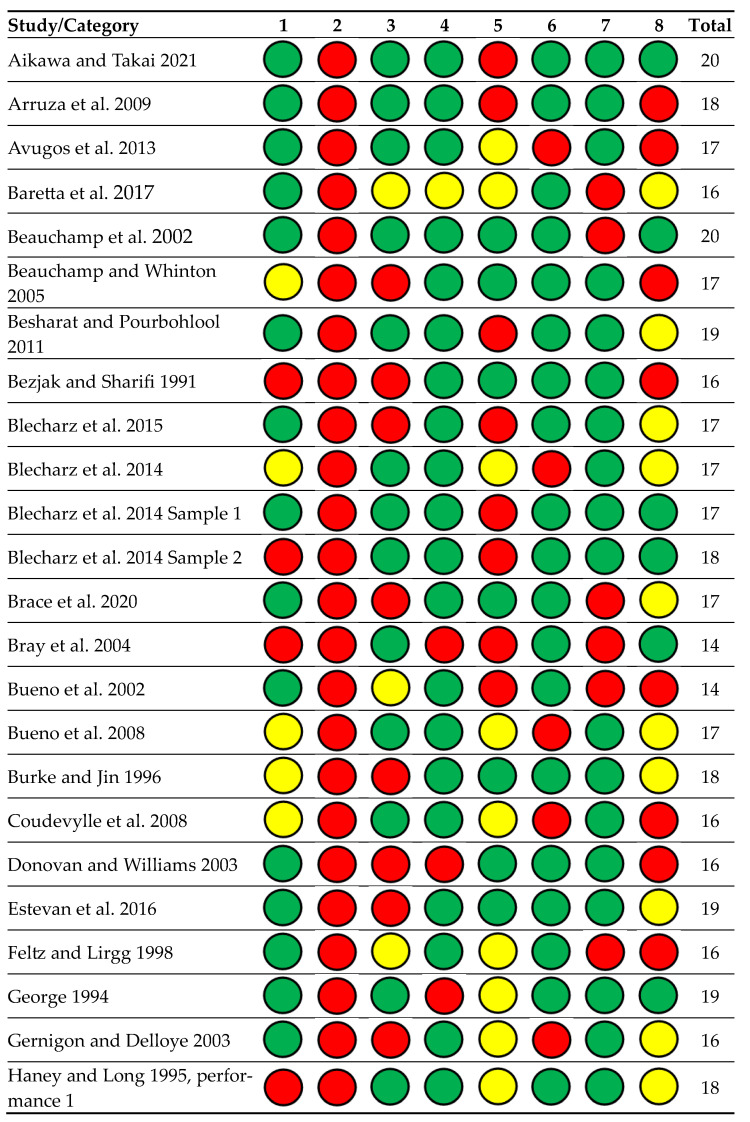

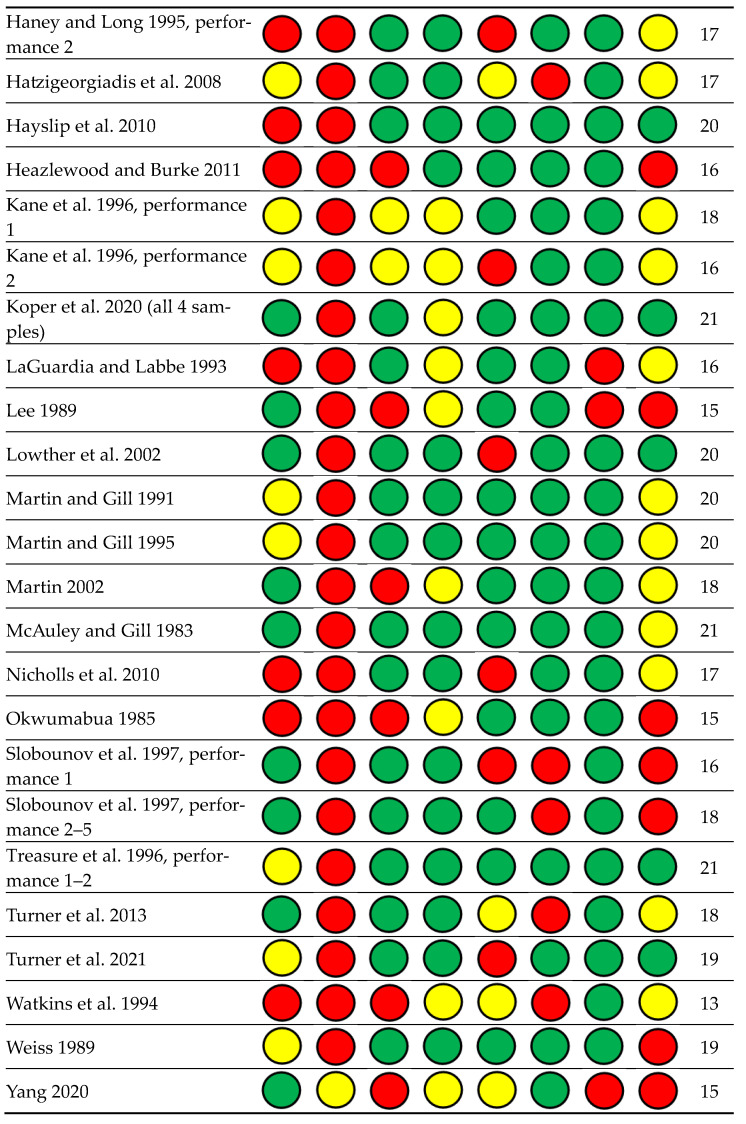
Risk of bias in included study data collection and participant selection methods. Categories explained: 1 = sample, 2 = recruitment, 3 = nonresponse, 4 = collection method, 5 = performance measure, 6 = setting, 7 = collection setting, and 8 = anonymity/confidentiality assured. Colors explained: green = 3, yellow = 2, and red = 1. References included in the figure [15,16,17,18,24,25,26,27,28,29,30,31,32,33,34,35,36,37,38,39,40,41,42,43,44,45,46,47,48,49,50,51,52,53,54,55,56,57,58,59,60,61,62,63].

Regarding our study quality score (see Figure 2 for details), the mean score (possible range of 9 to 27 points) was 17.63 (SD = 2.07) for our 55 samples with a median of 17 and a range from 13 points to 21 points. We examined the sample study quality with meta-regression (see Supplement Figure S1). The meta-regression R^2^ was 0.00 (computed value −0.06), indicating that no variance in the pre-event self-efficacy and sports performance relationship was explained by the study quality ratings. In Figure 2, the green circles represent number 3, the yellow circles represent number 2, and the red circles represent number 1.

### 3.2. Individual Study Data, Synthesis of Results, and Risk of Bias across Studies

**Figure 3:**
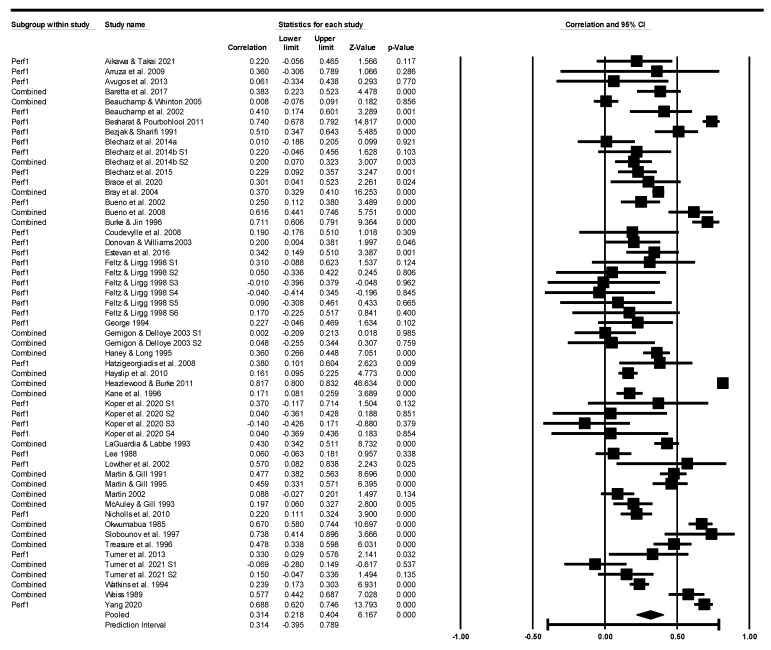
Sample effect size statistics expressed as correlations (*r*) and corresponding forest plots. References included in the figure [15,16,17,18,24,25,26,27,28,29,30,31,32,33,34,35,36,37,38,39,40,41,42,43,44,45,46,47,48,49,50,51,52,53,54,55,56,57,58,59,60,61,62,63].

Figure 3 contains individual data for all studies and the corresponding forest plot. The mean pre-event self-efficacy to sports performance effect size relationship was 0.31 with a 95% confidence interval of 0.22 to 0.40. The mean effect size, with criterion alpha of 0.05, was different from zero with a *Z*-value = 6.16, *p* < 0.001. The Q-test for heterogeneity, the test that analysed the studies (samples) that shared the same common effect size, was not supported (Q-value = 1491.58 with 54 degrees of freedom, *p* < 0.001). The true effect prediction interval was −0.39 to 0.79 (τ^2^ = 0.13, *I^2^* = 96%), suggesting that 95% of comparable studies would fall within this interval.

**Figure 4:**
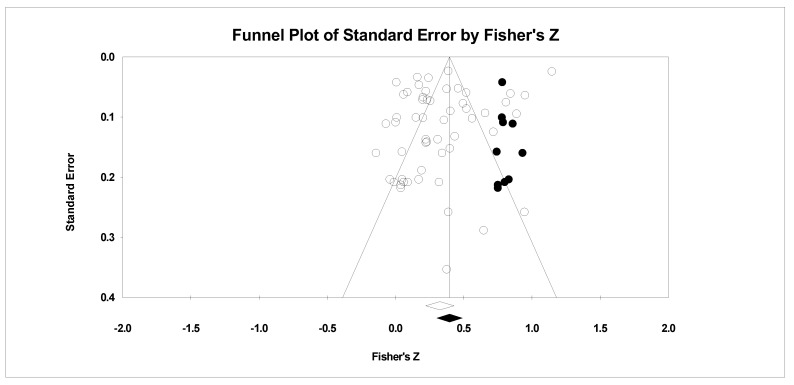
Random effects plot trimmed and filled. The open circles are the data points, and the filled circles are the result of the trim and fill analysis. The clear rhombus is the mean effect size, and the filled rhombus is the trim and filled mean effect size.

Concerning the risk of bias analyses, the classic fail-safe *n* (1-tail, *n* = 21,164) and Orwin’s *n* (*n* = 185) statistics both indicated that the pre-event self-efficacy and sports performance relationship evident in our included studies would require a large number of missed or future studies reporting no relationship for our effect size to be reduced to either non-significant or a small (*r* of 0.10) value. As presented in Figure 4, some bias existed in the data with the trim being 10 samples to the right using the random-effects model. This suggests that the published literature is an underrepresentation of the relationship between pre-event self-efficacy and sports performance (*r* = 0.38, 95% CI 0.29, 0.45). Last, while checking bias, we examined if the study quality biased our mean effect size with meta-regression. The meta-regression result indicated that the total study quality score had no meaningful relationship on the pre-event self-efficacy and sports performance relationship (*R^2^* = −0.04, coefficient = 0.00, SE = 0.00, 95% CI −0.00, 0.00, *Z* = 0.79, *p* > 0.05).

### 3.3. Additional Sensitivity Analyses

**Figure 5:**
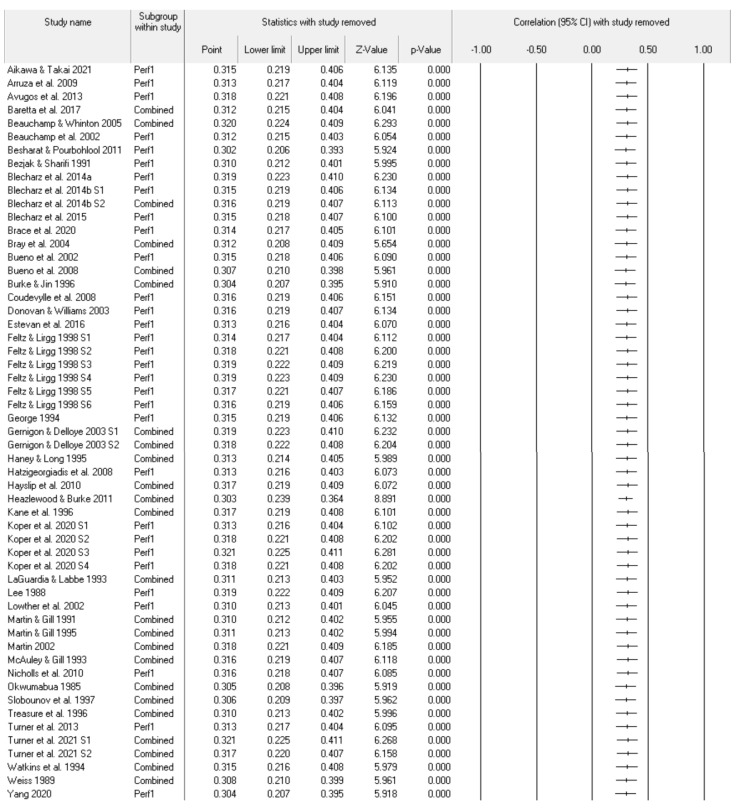
Random-effects model statistics and forest plots, with one study removed. References included in the figure [15,16,17,18,24,25,26,27,28,29,30,31,32,33,34,35,36,37,38,39,40,41,42,43,44,45,46,47,48,49,50,51,52,53,54,55,56,57,58,59,60,61,62,63].

**Figure 6:**
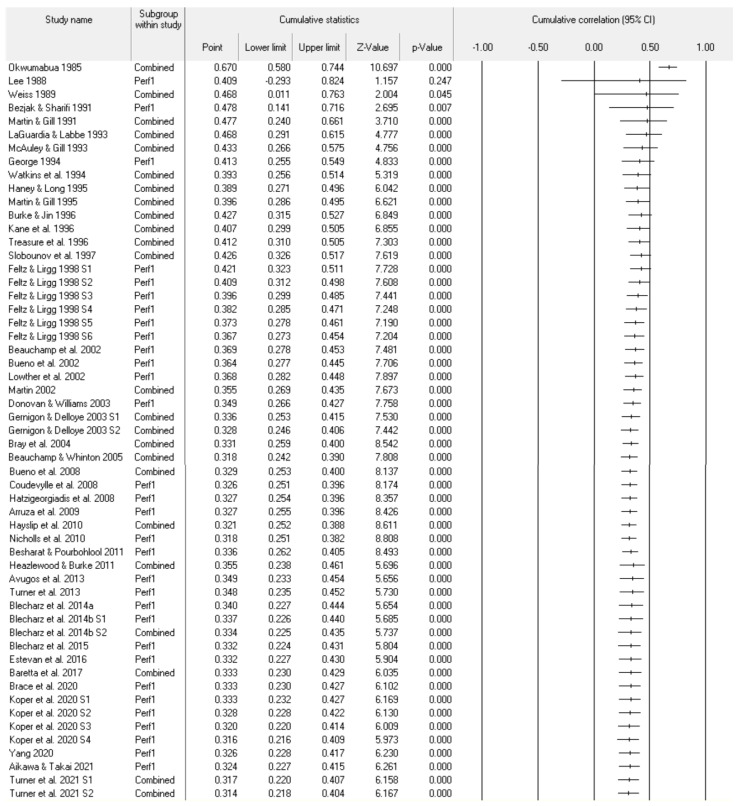
Random-effects model cumulative analysis statistics and forest plot by year. References included in the figure [15,16,17,18,24,25,26,27,28,29,30,31,32,33,34,35,36,37,38,39,40,41,42,43,44,45,46,47,48,49,50,51,52,53,54,55,56,57,58,59,60,61,62,63].

Figure 5 depicts the CMA remove-one analysis that gauges the impact of each included study. The individual point estimates appear very consistent (ranging from 0.30 to 0.32) and hence no one study unduly impacted the results. The forest plot is a visual representation of the consistency of the results. Figure 6 represents the CMA cumulative analysis program by year. Starting in 1983 until 2021, the cumulative analysis suggests some initial variation in the consistency of the pre-event self-efficacy and sports performance relationship. Since 2011, the relationship appears to have been very consistent in both point estimates and confidence limits.

### 3.4. Moderator Analyses

Table 3 shows the moderator codes used in our analyses and the results of the moderator analyses are shown in Table 4. Though nonsignificant at the traditional *p* < 0.05, examining the 95% confidence intervals and prediction intervals suggests that pre-event self-efficacy is more predictive (with *r* difference > 0.10) of sports performance in closed skill sports, with subjective performance measures, self-referenced performance measures, questionnaire and performance concordance, and elite athletes.

We examined sample sex make-up using meta-regression (see Supplementary Figure S2). The meta-regression *R*^2^ was 0.00 (computed value −0.04), indicating that no variance in the pre-event self-efficacy and sports performance relationship was explained by the sex of the study participants.

### 3.5. Certainty of Evidence

Some caution is needed to best interpret and provide certainty of evidence ratings, given the observed prediction interval values ranging from negative to positive. It is important to remember that the prediction interval is a range or bandwidth of plausible values that can include the true effect. Hence, we are certain that the overall pre-event self-efficacy and performance relationship is, at best, medium in magnitude, while it is plausible that in some cases the relationship may not support our mean effect size. For instance, new studies with objective performance in sports greater than 10 min in duration may result in a negative and large-magnitude relationship between pre-event self-efficacy and performance. This, of course, is speculative. Concerning our moderators, the evidence suggests with some certainty that measurement concordance, athlete level, and sports skill matter when investigating the self-efficacy and sports performance relationship. Last, of all the data points, certainty is the highest that self-rated performance will result in a positive, small relationship.

## 4. Discussion

The present study was a systematic review with meta-analysis of the published literature on the pre-event self-efficacy and sports performance relationship, which we conducted to fill a significant gap in the literature, given that two previous meta-analyses ostensibly on a very similar subject were either published more than 20 years ago and included non-sports studies [8] or included only a small fraction of primary studies that investigated pre-event self-efficacy and sports performance [11]. We distinguished the construct of self-efficacy from self-confidence based on the specific terminology used in our reviewed studies and also by cross-referencing our included studies against published self-confidence and performance meta-analyses to ensure that there was no overlap [13,72,73]. Overall, our results showed a robust, medium-sized effect of pre-event self-efficacy on sports performance, with the most important moderators being measurement concordance, type of sports skill, performance reference, and athlete level. Our findings mirrored those of previous meta-analyses on sports [8], work [9], and academic [10] domains. Our review addressed Borenstein and colleagues’ [22,23] concerns that cast new speculation on the certainty of our findings and those of past self-efficacy and performance reviews.

### 4.1. Moderator Effects

Considering the moderating variables individually, it is apparent that the pre-event self-efficacy and sports performance relationship is stronger in closed-skill sports than in open-skill sports. The relationship in closed-skill sports such as triathlon [38,47] and diving [15] was often large in magnitude, whereas in open-skill sports such as ice hockey [42] and cricket [60], the relationship was typically minimal to moderate. As Terry [14] explained, when the direct influence of opponents on performance is minimized or eliminated, the true influence of psychological variables such as self-efficacy on performance is more readily identified.

Similarly to the measure of performance, the relationship between pre-event self-efficacy and sports performance tends to be stronger when performance is assessed subjectively by self or coach rating [30,63] rather than assessed by win/loss records or objective scores [41,46]. This can be explained by the fact that objective measures do not always capture the quality of performance of the individual concerned [14]. For example, an athlete may perform exceptionally well but ultimately lose to a more talented opponent, which would be rated objectively as a loss but subjectively as a good performance.

The notion of concordance between self-efficacy and performance measures was also shown to be an important moderator. For example, studies in which the self-efficacy of achieving a personal goal, such as finishing a triathlon [18] or a marathon [57] at a particular time, was correlated against actual finish time were highly concordant. Such studies tended to produce larger effects than studies where the self-efficacy measure and the performance measure were nonconcordant, such as where participants rated their self-efficacy for sports in general but performance was assessed in, for example, specific boccia competitions [49].

The elite versus the non-elite status of the athlete was another variable shown to be an important moderator of the pre-event self-efficacy and sports performance relationship. The relationship reported tended to be stronger in studies of elite athletes [15,30] than in studies that used nonelite athletes as participants [40,60]. Elite athletes tend to display relative homogeneity of skill and fitness (i.e., they are all highly skilled and well-conditioned), whereas non-elite athletes tend to display a greater range of skill and fitness levels. In elite competitions, where important physical determinants of performance are evened out, psychological factors such as pre-event self-efficacy come to the fore, whereas at lower levels, the influence of psychological variables is masked by the relative heterogeneity of physical determinants of performance [14].

Contrary to the proposed moderating influence of sports duration [14], the relationship between pre-event self-efficacy and sports performance was somewhat stronger for longer duration (>10 min.) than shorter duration (<10 min.) sports. Terry proposed that pre-event psychological states, including self-efficacy, would be more closely related to performance in short-duration sports than in long-duration sports because the scope for psychological states to change after the contest had started would be greater if the sport lasted longer. Our findings indicate that this proposition does not hold true for self-efficacy, whereas it was supported in a meta-analysis of the literature on self-confidence and sports performance [13].

The relationship via meta-regression between pre-event self-efficacy and sports performance was shown to be unimportant for male and female sample make-ups. This finding does not align with the results of a meta-analysis summarizing the self-confidence and sports performance literature [13], which showed a stronger relationship for men than women. There is no obvious explanation for this inconsistency, which suggests that additional studies exploring sex and gender differences in self-efficacy and sports performance relationships are warranted.

It should be noted that in all instances, the trends described above did not reach statistical significance (the closest being for concordance at *p* = 0.08); therefore, statistical purists might argue that none of the variables we examined truly moderated the strength of the relationship between pre-event self-efficacy and sports performance. However, in the words of Andersen and Stoove [74], “the sanctity of *p* < 0.05 obfuscates good stuff”, and we contend that moderation effects of the type of sports and performance measure, level of athlete, and especially concordance between the self-efficacy and performance measures should be given due consideration by researchers when designing future studies.

### 4.2. Strengths, Limitations, Future Directions, and Practical Applications

A strength of our meta-analysis was the inclusion of 44 investigations of relationships specifically between pre-event self-efficacy and subsequent sports performance, spanning over five decades from the 1980s to the 2020s. Unlike previous related meta-analyses [8,11], we restricted our inclusion criteria to studies of sports events, excluding studies of physical education or fitness tasks. We focused on the pre-event assessment of self-efficacy and excluded post-event assessment, and we retained conceptual clarity by only including studies measuring self-efficacy rather than also including studies of self-confidence. A second strength was our comprehensive use of meta-analysis statistics, which included sensitivity analyses and the introduction of prediction interval statistics to this area of the literature. A third strength of our meta-analysis involved the assessment of moderators, the results of which will help guide future researchers and practitioners. As an example, moderator analyses showed that using a self-referenced performance criterion rather than an objective measure is more likely to reveal a positive relationship between pre-event self-efficacy and sports performance. Also, investigations in closed-skill sports and with elite athletes are more likely to reveal a positive relationship between self-efficacy and sports performance than those conducted in open-skill sports and with sub-elite athletes.

Limitations in the pre-event self-efficacy and sports performance literature result from non-uniform measures and different styles of self-efficacy measurement, vague reporting of pertinent details, and extensive use of convenience samples. As discussed in this review and other similar meta-analyses with pre-event measures [13,19,20], these issues are common. Overcoming such limitations would require a uniform reporting system to be adopted by all the journals. Even if such a lofty ambition is achieved, previously published literature cannot be changed, so these limitations are inevitable for the meta-analyst.

To conduct our systematic review with meta-analysis, we followed the PRISMA statement [21], which provides a step-by-step methodology with many specific elements. Despite following this structured approach, there are limitations in formulating and conducting such reviews. For instance, even with a thorough search of several relevant sources, the number of missed studies is unknown. Also, we attempted to include non-English studies to best represent the totality of the literature. Our search was conducted in English; thus, non-English studies of relevance were harvested typically where either a title, abstract, or keywords were written in English. To extract the relevant details of methodology and results, we then applied Google Translate, which we acknowledge may provide less than 100% accurate translation. However, although the use of Google Translate may be a potential limitation, this was balanced by our inclusion of studies without a language restriction, which can be seen as a methodological strength.

Another potential limitation relates to our categorization of athletes as elite or sub-elite. Although we applied the coding systems developed by Kyllo and Landers [75] and Swann et al. [76] to categorize athlete samples, we encountered great variation across studies in the terminology used to describe athletes of different ages and levels of competition. Finally, the risk of bias and overall quality of the study scores that we attributed to the studies may also represent a potential limitation. Locating all pertinent information in the primary studies was a painstaking and, at times, difficult process due to inconsistent reporting of methods and results. Few studies used random sampling procedures, and although we have confidence in our findings, we acknowledge that they are based on a body of literature that is generally limited in overall quality.

In terms of future directions in this area of research, we see much scope to improve the overall quality of self-efficacy and sports performance studies. With this aim in mind, we recommend more repeated-measures designs and cross-lagged designs that attempt to address issues of causality related to whether high self-efficacy improves performance, whether good performance improves self-efficacy, or whether a virtuous circle is evident whereby high self-efficacy leads to better performance, which further enhances self-efficacy, and so on. We also recommend a more random selection of participants and better reporting of details involving large data collections (e.g., at marathon events), including the number of people approached and the number who accepted. Another productive line of enquiry would be to focus on one or more moderator variables. For example, collecting both self and other referenced performance statistics and/or assessing both elite and non-elite participants in the same event. Large-scale events with both elite and non-elite competitors, such as marathons or triathlons, offer suitable environments for such research.

The key practical application of the findings is that the relationship between pre-event self-efficacy and sports performance is of sufficient strength and robustness to warrant athletes in all sports and at all levels, but especially those in closed-skill sports at an elite level, devoting time to activities specifically designed to enhance self-efficacy. There are many sports psychology self-help books [77,78,79,80] that include activities and exercises to promote self-efficacy that athletes, coaches, and sports psychology practitioners may find beneficial.

## 5. Conclusions

The relationship between pre-event self-efficacy and sports performance is medium in size and robust from 1983 to 2021 across a wide variety of sports and athletes at all levels. The pre-event self-efficacy and sports performance relationship varied little over time, and no one study impacted the relationship unduly. For researchers investigating pre-event self-efficacy as a predictor of sports performance, considering the concordance between the self-efficacy measure and sports performance measure is important, as are other moderator variables common to the self-efficacy and sports performance literature. Practitioners should be aware that higher pre-event self-efficacy does not always result in better sports performance. However, evidence suggests that pre-event self-efficacy has a meaningful impact on sports performance and hence coaches, sports psychology practitioners, and athletes themselves should work at maximizing athlete self-belief to perform and thus achieve in competitive sports contests.

## Figures and Tables

**Table 1:** Individual study bias questions and rating explanations summed to a study quality score.

		Rating Explanations	
Bias	Low Risk (3 Points)	Medium Risk (2 Points)	High Risk (1 Point)
Sample	Elite or advanced	Intermediate or youth	Mix
Recruitment	Stated random selection occurred from a much larger group (e.g., from all athletes at an event)	Random selection occurred within a group of athletes (e.g., a college team at an event)	No random selection of any kind stated
Nonresponse	Appears most participants completed the measures	Participants recruited; coaches/parents denied participation in at least 25%	Many did not do it (e.g., a big race, subjects recruited at the race, most likely most did not do it)
Collection setting	Administered in person	Mix in person and unsupervised	Unsupervised (e.g., survey left in lockers) or mail, online
Performance measure	Event time, win–loss, placement, event scores	Participant statistics	Subjective
Setting	Actual event	No medium rating	Contrived
Collection consistency	All the same	No medium rating	A mix of ways (e.g., individual for some, in large groups for others)
Anonymity	Yes, stated	Not stated but informed consent presented	Not stated

**Table 2:** Study characteristics.

Study	Age Means, Ranges, or Descriptions	N	% Male	Ctry.	Sport	Performance Measure
Aikawa and Takai [24]	19.70	52	56.76	JP	Gymnastics	Performance statistics
Arruza et al. [25]	23.60	11	72.73	US	Mix individual	Self-evaluation
Avugos et al. [26]	19.74	26	100	IL	Basketball	Shots made percentage
Baretta et al. [27]	39.79	129	66.67	IT	Freediving	Performance statistics
Beauchamp et al. [28]	20.80	60	100	CA	Golf	Event score
Beauchamp and Whinton [29]	12.48	187	20.32	UK	Equestrian	Performance statistics
Besharat and Pourbohlool [30]	23.03	246	60.57	IR	Mix individual and team	Coach evaluation
Bezjak and Sharifi [31]	34.80	98	56.64	US	Marathon	Event time
Blecharz et al. [32]	21.77	197	57.36	US	Mix	Performance satisfaction
Blecharz et al. [33]	18.14	101	100	PL	Soccer	Shots made percentage
Blecharz et al. (2 samples) [34]	22.70, 18.23	56, 113	36.00, 100	US	Mix, soccer	Performance satisfaction
Brace et al. [35]	38.86	56	67.85	Mix	Ultra-endurance	Event time
Bray et al. [36]	14.80	295	100	ES	Soccer	Self-evaluations
Bueno et al. [18]	31.01	90	?	**ES**	Triathlon	Event times
Bueno et al. [37]	22.00	35	0	US	Track and field (endurance)	Performance statistics (meters covered in 5 min)
Burke and Jin [38]	NR	40	92.5	AU	Triathlon	Event times
Coudevylle et al. [39]	20.22	31	51.61	FR	Basketball	Performance statistics scale (shots and passes)
Donovan and Williams [40]	17.00 to 21.00	100	60.5	US	Track and field	Mercier scoring of performance
Estevan et al. [41]	22.03	86	55.81	**ES**	Taekwondo	Winners vs. losers
Feltz and Lirgg [42]	19.00 to 22.00	159 (6 teams)	NR	US	Ice hockey	Game statistics including winning percentage
George [43]	17.30, 20.70	28, 25	100	US	Baseball	Hitting statistics
Gernigon and Delloye [17]	19.90	62	67.74	FR	Running sprints	International Amateur Athletic Federation points earned based on 60 m trial
Haney and Long [44]	19.16	178	0	US	Mix team	Shots scored scale
Hatzigeorgiadis et al. [45]	13.26	46	46	GR	Tennis	Forehand drive score
Hayslip et al. [46]	44.46	220	100	US	Golf	Event score
Heazlewood and Burke [47]	32.40, 33.60	416	NR	AU	Triathlon	Event time
Kane et al. [48]	Adolescents	216, 204	100	US	Wrestling	Win/loss records
Koper et al. [49] (4 samples)	33.90, 28.10, 29.00, 29.90	18, 25, 42, 24	78.0, 80.0, 74.0, 83.0	Mix	Boccia	Event results
LaGuardia and Labbe [50]	Adults	63	68.25	US	Running	Pace times
Lee [51]	21.00	257	17.9	US	Mix individual and team	Team winning percentage
Lowther et al. [16]	19.00 to 28.00	15	100	UK	Soccer	Self-referenced measure
Martin and Gill [52]	16.00	73	100	US	Track and field	Event time, placement
Martin and Gill 1995 [53]	16.00	86	55.81	US	Track and field	Event time, placement
Martin [54]	35.40	51	100	US	Wheelchair road racing	Event time, placement
McAuley and Gill [55]	19.00 to 22.00	52	0	US	Gymnastics	Event scores
Nicholls et al. [56]	21.30	307	82.08	UK	Mix individual and team	Performance rating
Okwumabua [57]	35.50	90	91.11	US	Marathon	Best 10K time
Slobounov et al. [15]	18.00 to 21.00	6	50	US	Diving	Self-evaluation
Treasure et al. [58]	16.03	70	100	US	Wrestling	Winning percentage, point statistics
Turner et al. [59]	16.45	42	100	UK	Cricket	Batting test statistics
Turner et al. [60]	13.26	92	0	UK	Netball	Coach evaluation
Watkins et al. [61]	12.50	205	100	US	Baseball	Hitting statistics
Weiss [62]	11.50	22	100	US	Gymnastics	Event scores
Yang [63]	NR	270	NR	ES	Basketball	Team performance score

Bold country abbreviation = study written in non-English. Abbreviations: Country (Crty.), not reported (NR); Australia (AU), Canada (CA), Spain (ES), France (FR), Greece (GR), Israel (IL), Islamic Republic of Iran (IR), Italy (IT), Japan (JP), Poland (PL), United Kingdom (UK), and United States of America (US). ? = not enough information presented to decide.

**Table 3:** Moderator coding.

Study	Duration	Skill	Event	Measure	Reference	Concordance	Level
Aikawa and Takai [24]	>10	C	ACT	SUB	Self	Yes	Sub
Arruza et al. [25]	<10, >10	C, O	ACT	SUB	Self	Yes	Elite
Avugos et al. [26]	<10	C	CON	OBJ	OTH	Yes	Elite
Baretta et al. [27]	<10	C	ACT	OBJ	OTH	Yes	Elite
Beauchamp et al. [28]	>10	C	ACT	OBJ	OTH	Yes	Sub
Beauchamp and Whinton [29]	<10, >10	C	ACT	OBJ	OTH	Yes	Sub
Besharat and Pourbohlool [30]	<10, >10	C, O	ACT	SUB	OTH	Yes	Elite
Bezjak and Sharifi [31]	>10	C	ACT	OBJ	OTH	No	Mix
Blecharz et al. [32]	<10, >10	C, O	ACT	SUB	Self	No	Elite
Blecharz et al. [33]	<10	C	CON	OBJ	OTH	No	Sub
Blecharz et al. [34]	>10	O	ACT	SUB	Self	No	Sub
Brace et al. [35]	>10	C	ACT	OBJ	OTH	No	Elite
Bray et al. [36]	>10	O	ACT	SUB	Self	Yes	Sub
Bueno et al. [18]	>10	C	ACT	OBJ	Self	Yes	Sub
Bueno et al. [37]	<10	C	CON	OBJ	OTH	Yes	Sub
Burke and Jin [38]	>10	C	ACT	OBJ	OTH	Yes	Sub
Coudevylle et al. [39]	<10	C	CON	OBJ	OTH	Yes	Sub
Donovan and Williams [40]	<10	C	ACT	OBJ	OTH	Yes	Sub
Estevan et al. [41]	<10	O	ACT	OBJ	OTH	No	Sub
Feltz and Lirgg [42]	>10	O	ACT	OBJ	OTH	No	Sub
George [43]	>10	O	ACT	OBJ	OTH	Yes	Sub
Gernigon and Delloye [17]	<10	C	CON	OBJ	OTH	Yes	Sub
Haney and Long [44]	<10	C	ACT	OBJ	OTH	Yes	Sub
Hatzigeorgiadis et al. [45]	<10	C	CON	OBJ	OTH	Yes	Sub
Hayslip et al. [46]	>10	C	ACT	OBJ	OTH	No	Sub
Heazlewood and Burke [47]	>10	C	ACT	OBJ	OTH	Yes	Sub
Kane et al. [48]	<10	O	ACT	SUB	Self	No	Sub
Koper et al. [49] (4 samples)	<10	O	ACT	OBJ	OTH	No	Sub
LaGuardia and Labbe [50]	<10, >10	C	ACT	OBJ	OTH	Both	Sub
Lee [51]	<10, >10	C, O	ACT	OBJ	OTH	Yes	Sub
Lowther et al. [16]	>10	O	ACT	SUB	Self	Yes	Elite
Martin and Gill [52]	<10	C	ACT	OBJ	OTH	Yes	Sub
Martin and Gill [53]	>10	C	ACT	OBJ	OTH	Yes	Sub
Martin [54]	>10	C	ACT	OBJ	OTH	Yes	Elite
McAuley and Gill [55]	<10	C	ACT	OBJ	OTH	Yes	Sub
Nicholls et al. [56]	?	C, O	ACT	SUB	Self	No	Sub
Okwumabua [57]	>10	C	ACT	OBJ	OTH	Yes	Sub
Slobounov et al. [15]	<10	C	CON	SUB	Self	Yes	Elite
Treasure et al. [58]	<10	O	ACT	OBJ	OTH	Both	Sub
Turner et al. [59]	<10	C	CON	OBJ	OTH	Yes	Elite
Turner et al. [60]	>10	O	ACT	OBJ/SUB	OTH	Yes	Sub
Watkins et al. [61]	<10	C	CON	OBJ	OTH	Yes	Sub
Weiss [62]	<10	C	ACT	OBJ	OTH	Yes	Sub
Yang [63]	>10	O	ACT	SUB	OTH	No	Sub

Abbreviations: L = low, M = medium, and H = high; ? = not enough information presented to decide, O = open skill sport, C = closed skill sport, OBJ = objective, SUB = subjective-referenced performance, OTH = other-referenced performance, Self = performance self-referenced, and Sub = sub-elite athlete level.

**Table 4:** Moderator results.

Moderator	Group	k	*r*	95% CI	95% PI	Q	*p*-Value	τ^2^	*I^2^*
Sport duration	<10	24	0.27	0.19, 0.35	−0.13, 0.60			0.04	83.07
	>10	25	0.34	0.17, 0.49	−0.51, 0.85	0.52	0.47	0.19	97.63
Sport skill	Closed	29	0.37	0.22, 0.50	−0.48, 0.86			0.19	97.62
	Open	21	0.23	0.12, 0.33	−0.23, 0.61	2.21	0.13	0.05	85.27
Event	Actual	45	0.32	0.21, 0.42	−0.42, 0.81			0.14	96.86
	Contrived	10	0.26	0.12, 0.40	−0.22, 0.64	0.44	0.50	0.04	93.70
Performance measure	Objective	42	0.29	0.16, 0.41	−0.52, 0.82			0.18	96.82
	Subjective	11	0.38	0.23, 0.52	−0.22, 0.78	0.95	0.33	0.07	93.70
Performance reference	Other	44	0.31	0.19, 0.43	−0.50, 0.83			0.18	96.89
	Self	10	0.25	0.18, 0.33	0.02, 0.46	0.65	0.42	0.01	67.72
Concordance	Yes	32	0.37	0.23, 0.49	−0.44, 0.85			0.17	97.44
	No	21	0.22	0.11, 0.32	−0.23, 0.59	2.98	0.08	0.05	85.08
Athlete level	Elite	10	0.40	0.17, 0.59	−0.44, 0.87			0.13	92.19
	Sub-elite	42	0.28	0.16, 0.39	−0.46, 0.79	0.93	0.33	0.15	96.92

Abbreviation: k = number of samples, *r* = mean random-effect modeled effect size, CI = confidence interval, PI = prediction interval, τ^2^ = Tau-squared, and I^2^ = I-squared.

## Data Availability

All data are contained in the article tables.

## Supplementary Materials

The following supporting information can be downloaded at: https://www.mdpi.com/article/10.3390/sports11110222/s1, Supplementary Table S1. PRISMA Checklist, Supplementary Figure S1. Study quality meta-regression, Supplementary Figure S2. Percent male meta-regression, and Supplementary Table S2. Search strategy.

## Review Registration

Review not registered.
