# Supplementary material for: Pre-Event Self-Efficacy and Sports Performance: A Systematic Review with Meta-Analysis
Source: Sports (Basel). 2023 Nov 12;11(11):222. doi: 10.3390/sports11110222 (PMC10675036; doi:10.3390/sports11110222)
Supplement: Supplementary file 1 [file sports-11-00222-s001.zip › sports-2670105-supplementary.pdf]

Supplemental Content Order

Supplement Table S1. PRISMA 2020 Checklist

Supplement Figure S1. Meta-regression plot of the relationship of study quality for each study sample and the pre-event self-efficacy and sport performance relationship

Supplement Figure S2. Meta-regression plot of the relationship of percent male in each study sample and the pre-event self-efficacy and sport performance relationship.

Supplement Table S2. Search Results

Supplement Table S1. PRISMA 2020 Checklist

Section and Topic	Item #	Checklist item	Location where item is reported
**TITLE**			
Title	1	Identify the report as a systematic review.	Title
**ABSTRACT**			
Abstract	2	See the PRISMA 2020 for Abstracts checklist.	Yes, within word limits.
**INTRODUCTION**			
Rationale	3	Describe the rationale for the review in the context of existing knowledge.	Introduction
Objectives	4	Provide an explicit statement of the objective(s) or question(s) the review addresses.	1.2.
**METHODS**			
Eligibility criteria	5	Specify the inclusion and exclusion criteria for the review and how studies were grouped for the syntheses.	2.1.
Information sources	6	Specify all databases, registers, websites, organisations, reference lists and other sources searched or consulted to identify studies. Specify the date when each source was last searched or consulted.	2.2.
Search strategy	7	Present the full search strategies for all databases, registers and websites, including any filters and limits used.	2.2., supplement table, fig.1
Selection process	8	Specify the methods used to decide whether a study met the inclusion criteria of the review, including how many reviewers screened each record and each report retrieved, whether they worked independently, and if applicable, details of automation tools used in the process.	2.1.
Data collection process	9	Specify the methods used to collect data from reports, including how many reviewers collected data from each report, whether they worked independently, any processes for obtaining or confirming data from study investigators, and if applicable, details of automation tools used in the process.	2.3.
Data items	10a	List and define all outcomes for which data were sought. Specify whether all results that were compatible with each outcome domain in each study were sought (e.g. for all measures, time points, analyses), and if not, the methods used to decide which results to collect.	2.3.
	10b	List and define all other variables for which data were sought (e.g. participant and intervention characteristics, funding sources). Describe any assumptions made about any missing or	2.3.

Section and Topic	Item #	Checklist item	Location where item is reported
		unclear information.	
Study risk of bias assessment	11	Specify the methods used to assess risk of bias in the included studies, including details of the tool(s) used, how many reviewers assessed each study and whether they worked independently, and if applicable, details of automation tools used in the process.	2.4.
Effect measures	12	Specify for each outcome the effect measure(s) (e.g. risk ratio, mean difference) used in the synthesis or presentation of results.	2.5.
Synthesis methods	13a	Describe the processes used to decide which studies were eligible for each synthesis (e.g. tabulating the study intervention characteristics and comparing against the planned groups for each synthesis (item #5)).	2.5.
	13b	Describe any methods required to prepare the data for presentation or synthesis, such as handling of missing summary statistics, or data conversions.	2.5.
	13c	Describe any methods used to tabulate or visually display results of individual studies and syntheses.	2.5.
	13d	Describe any methods used to synthesize results and provide a rationale for the choice(s). If meta-analysis was performed, describe the model(s), method(s) to identify the presence and extent of statistical heterogeneity, and software package(s) used.	2.5.
	13e	Describe any methods used to explore possible causes of heterogeneity among study results (e.g. subgroup analysis, meta-regression).	2.5.
	13f	Describe any sensitivity analyses conducted to assess robustness of the synthesized results.	2.5.
Reporting bias assessment	14	Describe any methods used to assess risk of bias due to missing results in a synthesis (arising from reporting biases).	2.5.
Certainty assessment	15	Describe any methods used to assess certainty (or confidence) in the body of evidence for an outcome.	2.5.
**RESULTS**			
Study selection	16a	Describe the results of the search and selection process, from the number of records identified in the search to the number of studies included in the review, ideally using a flow diagram.	3.1.
	16b	Cite studies that might appear to meet the inclusion criteria, but which were excluded, and explain why they were excluded.	3.1.
Study characteristics	17	Cite each included study and present its characteristics.	3.1.

Section and Topic	Item #	Checklist item	Location where item is reported
Risk of bias in studies	18	Present assessments of risk of bias for each included study.	3.2.
Results of individual studies	19	For all outcomes, present, for each study: (a) summary statistics for each group (where appropriate) and (b) an effect estimate and its precision (e.g. confidence/credible interval), ideally using structured tables or plots.	3.2.
Results of syntheses	20a	For each synthesis, briefly summarise the characteristics and risk of bias among contributing studies.	3.2.
	20b	Present results of all statistical syntheses conducted. If meta-analysis was done, present for each the summary estimate and its precision (e.g. confidence/credible interval) and measures of statistical heterogeneity. If comparing groups, describe the direction of the effect.	3.2.
	20c	Present results of all investigations of possible causes of heterogeneity among study results.	3.2., 3.4.
	20d	Present results of all sensitivity analyses conducted to assess the robustness of the synthesized results.	3.2., 3.3.
Reporting biases	21	Present assessments of risk of bias due to missing results (arising from reporting biases) for each synthesis assessed.	3.2.
Certainty of evidence	22	Present assessments of certainty (or confidence) in the body of evidence for each outcome assessed.	3.5.
**DISCUSSION**			
Discussion	23a	Provide a general interpretation of the results in the context of other evidence.	Discussion, 4.1.
	23b	Discuss any limitations of the evidence included in the review.	4.2.
	23c	Discuss any limitations of the review processes used.	4.2
	23d	Discuss implications of the results for practice, policy, and future research.	Conclusions and across discussion
**OTHER INFORMATION**			
Registration and protocol	24a	Provide registration information for the review, including register name and registration number, or state that the review was not registered.	Not registered.
	24b	Indicate where the review protocol can be accessed, or state that a protocol was not prepared.	All information provided in manuscript.
	24c	Describe and explain any amendments to information provided at registration or in the protocol.	None to report.

Section and Topic	Item #	Checklist item	Location where item is reported
Support	25	Describe sources of financial or non-financial support for the review, and the role of the funders or sponsors in the review.	Reported in funding statement.
Competing interests	26	Declare any competing interests of review authors.	No competing interests
Availability of data, code and other materials	27	Report which of the following are publicly available and where they can be found: template data collection forms; data extracted from included studies; data used for all analyses; analytic code; any other materials used in the review.	Supplement files and manuscript tables

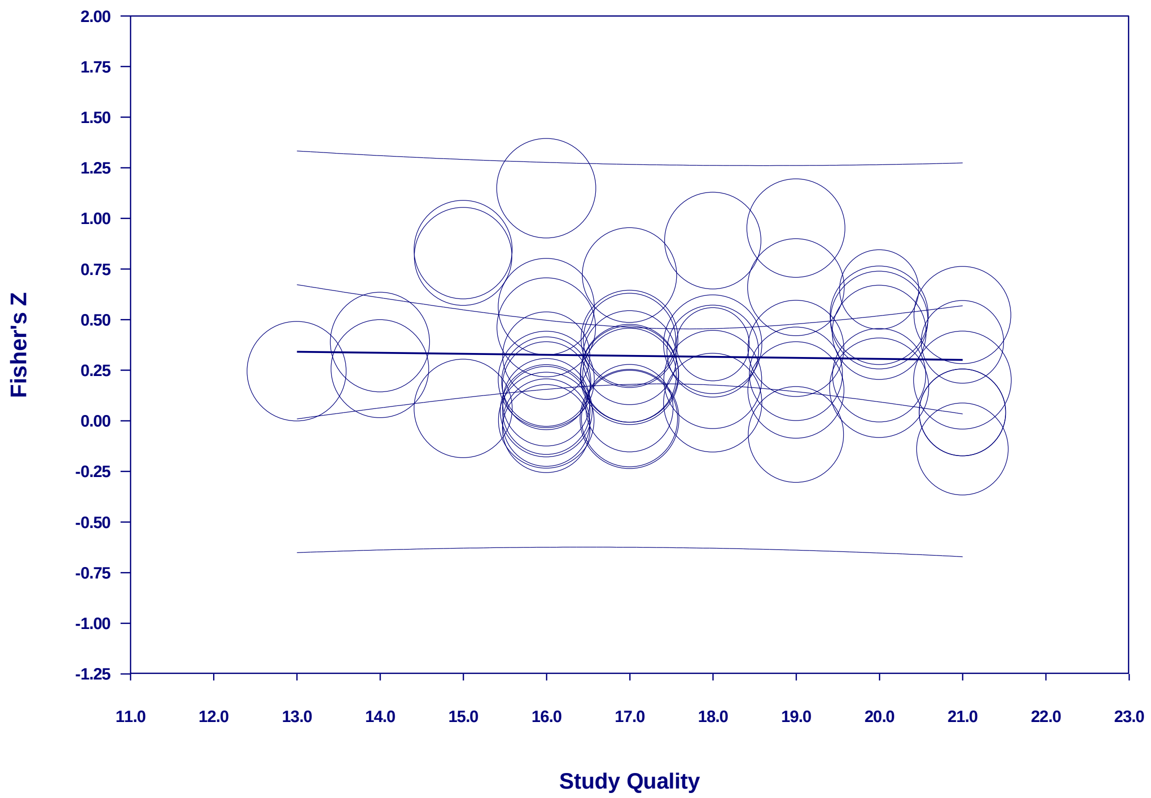

Supplement Figure S1. Meta-regression plot of the relationship of study quality for each study sample and the pre-event self-efficacy and sport performance relationship. Larger circles represent studies with more participants. The middle line is the regression line, the upper and lower lines are the 95% confidence intervals, and the second set (widest) upper and lower lines are the 95% prediction intervals.

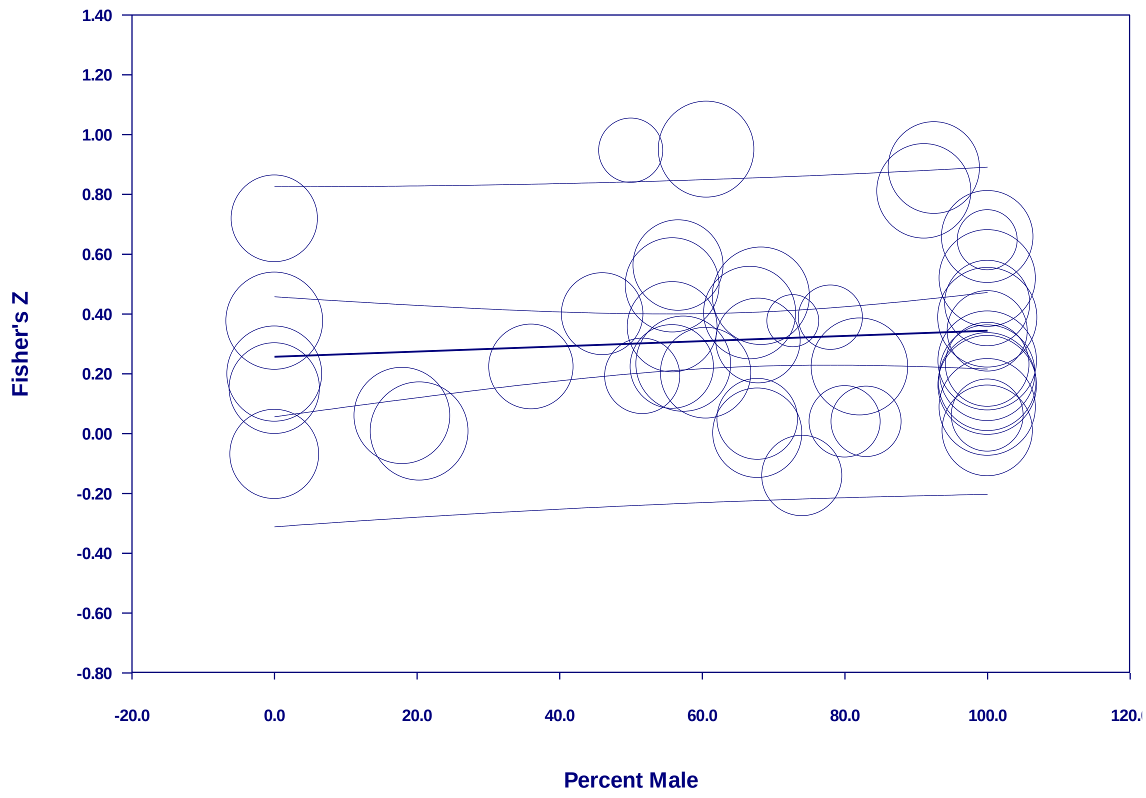

Supplement Figure S2. Meta-regression plot of the relationship of percent male in each study sample and the pre-event self-efficacy and sport performance relationship. Larger circles represent studies with more participants. The middle line is the regression line, the upper and lower lines are the 95% confidence intervals, and the second set (widest) upper and lower lines are the 95% prediction intervals.

Supplement Table S2. Search results.

Source		Citation
Moritz	1	Barling, J., & Abel, M. (1983). Self-efficacy beliefs and tennis performance. Cognitive Therapy and Research, 7(3), 265–272. https://doi.org/10.1007/BF01205140
Moritz	2	Barnes, M. W., Sime, W., Dienstbier, R. A., & Plake, B. (1986). A test of construct validity of the CSAI-2 questionnaire on male elite college swimmers. International Journal of Sport Psychology, 17(5), 364–374.
Moritz	3	Bezjak JE, Langga-Sharifi E. Validation of the Physical fitness opinion questionnaire against marathon performance. Perceptual & Motor Skills. 1991;73:993-994. doi:10.2466/PMS.73.7.993-994
Moritz	4	Burton D. Do anxious swimmers swim slower? Reexamining the elusive anxiety-performance relationship. Journal of Sport & Exercise Psychology. 1988;10(1):45-61. doi:10.1123/jsep.10.1.45
Moritz	5	Caruso CM, Dzewaltowski DA, Gill DL. Psychological and physiological changes in competitive state anxiety during noncompetition and competitive success and failure. Journal of Sport & Exercise Psychology. 1990;12:6-20. Accessed March 2, 2022. https://search-ebscohost-com.lib-e2.lib.ttu.edu/login.aspx?direct=true&db=ofm&AN=508374807&site=ehost-live
Moritz	6	Ewart CK, Stewart KJ, Gillilan RE, et al. Usefulness of self-efficacy in predicting overexertion during programmed exercise in coronary artery disease. The American journal of cardiology. 1986;57(8):557-561. doi:10.1016/0002-9149(86)90834-9
Moritz	7	Ewart CK, Stewart KJ, Gillilan RE, Kelemen MH. Self-efficacy mediates strength gains during circuit weight training in men with coronary artery disease. Medicine & Science in Sports & Exercise. 1986;18(5):531-540. doi:10.1249/00005768-198610000-00007
Moritz	8	Feltz DL. Path Analysis of the Causal Elements in Bandura's Theory of Self-Efficacy and an Anxiety-Based Model of Avoidance Behavior. Journal of Personality & Social Psychology. 1982;42(4):764-781. doi:10.1037/0022-3514.42.4.764
Moritz	9	Feltz, D. L. (1988). Gender differences in the causal elements of self-efficacy on a high avoidance motor task. Journalof Sportand Exercise Psychology, 70, 151-166.
Moritz	10	Feltz, D. L., Landers, D. M., & Raeder, U. (1979). Enhancing Self-efficacy in High-avoidance Motor Tasks: A Comparison of Modeling Techniques, Journal of Sport Psychology, 1(2), 112-122. Retrieved Mar 2, 2022

Moritz	11	Feltz DL, Mugno DA. A Replication of the Path Analysis of the Causal Elements in Bandura's Theory of Self-efficacy and the Influence of Autonomic Perception. Journal of Sport Psychology. 1983;5(3):263-277. Accessed March 2, 2022. https://search-ebscohost-com.lib-e2.lib.ttu.edu/login.aspx?direct=true&db=s3h&AN=20724802&site=ehost-live
Moritz	12	Feltz, D. L., & Riessinger, C. A. (1990). Effects of in vivo emotive imagery and performance feedback on self-efficacy and muscular endurance. Journalof SportPsychology, 72, 132-143.
Moritz	13	Garcia, A.w., & King, A. C. (1991). Predicting long-term adherence to aerobic exercise: A comparison of two models. Journalof SportandExercise Psychology, 73,394-410.
Moritz	14	George, T. R (1994). Self-confidence and baseball performance: A causal examination of self-efficacy theory. Journalof Sportand Exercise Psychology, 76,381-399.
Moritz		George 1994 sample 2
Moritz	15	George, T. R, Feltz, D. L., &Chase, M. A. (1992. Effects of model similarity on self-efficacy and muscular endurance: A second look. Journalof SportandExercise Psychology, 74, 237-248.
Moritz	16	Gould, D., & Weiss, M. R (1981). The effects of model similarity and model talk on self-efficacy and muscular endurance. Journal of SportPsychology, 3, 17-29.
Moritz	17	Kane, T. D., Marks, M. A, Zaccaro, S. J., & Blair, V. (1996). Selfefficacy, personal goals, and wrestlers' self-regulation. Journalof SportandExercise Psychology, 78, 36-48.
Moritz	18	Kaplan, R M., Ries, A. L., Prewitt, L. M.,&Eakin, F. (1994). Selfefficacyexpectations predictsurvival for patients with chronic obstructive pulmonary disease. Health Psychology, 73,366-368.
Moritz	19	Kavanagh, R M.,& Hausfeld, S. (1986). Physical performance and self-efficacy under happy and sad moods. Journalof Sport Psychology, 8, 112-123.
Moritz	20	Krane, V., & Williams, J. (1987). Performance and somatic anxiety, cognitive anxiety and confidence changes priorto competition. Journalof SportBehavior, 70, 47-56.
Moritz	21	LaGuardia, R., & Labbe, E. E (1993). Self-efficacy and anxiety and their relationship to training and race performance. Perceptual andMotor Skills, 77, 27-34.
Moritz	22	Lee, C. (1988). The relationship between goal setting, self-efficacy, and female field hockey team performance. International Journal of SportPsychology, 20, 147-161.
Moritz	23	Lerner, B.S., &Locke, EA (1995). The effects of goal setting, selfefficacy, competition and personal traits on performance

		of an endurance task. Journalof SportandExercise Psychology, 77, 138-152.
Moritz	24	Martin,J. J. & Gill, D. L. (1991. The relationships among competitive orientation, sport-confidence, self-efficacy, anxiety, and performance. Journal of Sport andExercise Psychology, 73, 149--159.
Moritz	25	Martin, J. J.,& Gill, D. L (1995). The relationships of competitive orientations and self-efficacy to goal importance, thoughts, and performance in high school distance runners. Journal of Applied Sport Psychology, 7,50-62.
Moritz	26	Maynard, I.w., &Howe, B. L. (1987). Interrelations oftrait and state anxiety with game performance of rugby players. Perceptual andMotor Skills, 64, 599--602.
Moritz	27	McAuley, E (1985). State anxiety: Antecedent or resultof sport performance? Journal of SportBehavior, 8, 71-77.
Moritz	28	McAuley, E. (1985). Modelling and self-efficacy: A test of Bandura's model. Journal of SportPsychology, 7, 283-295.
Moritz	29	McAuley, E. (1992. The role of efficacy cognitions in the prediction of exercise behavior in middle-aged adults. Journal of Behavioral Medicine, 75, 65-88.
Moritz	30	McAuley, E(1993). Self-efficacy and the maintenance of exercise participation in older adults. Journalof Behavioral Medicine, 76, 103-113.
Moritz	31	McAuley, E., & Courneya, K. S. (1992). Self-efficacy relationships with affective and exertion responses to exercise. Journalof Applied SocialPsychology, 22, 312-326.
Moritz	32	McAuley, E., & Courneya, K. S., & lettunich, J. (1991). Effects of acute and long-term exercise on self-efficacy responses in sedentary, middle-aged males and females. The Gerontologist, 37, 534-542.
Moritz	33	McAuley, E., & Gill, D. l. (1983. Reliability and validity of the physical self-efficacy scale in a competitive sportsetting. Journal of SportPsychology, 5, 410-418.
Moritz	34	McCullagh, P. (1987. Model similarity effects on motor performance. Journalof SportPsychology, 9, 249-260.
Moritz	35	Miller, J. T, & McAuley, E. (1987). Effects of a goal-setting training program on basketball free-throw self-efficacy and performance. The SportPsychologist, 7, 103-113.
Moritz	36	Murphy, S.M.,&Woolfolk, R. l. (1987. The effects of cognitive interventions on competitive anxiety and performance on a fine motor skill accuracy task. International Journalof SportPsychology, 78,152-166.
Moritz	37	Okwumabua,1 M. (1985). Psychological and physical contributionsto marathon performance: An exploratory investigation. Journalof SportBehavior, 8,163-171.

Moritz	38	Taylor, C. B., Bandura, A., Ewart, C. K., Miller, N. H., & DeBusk, R. F. (1985). Exercise testing to enhance wives confidence intheir husbands' cardiac capability soon afterclinically uncomplicated acute myocardial infarction. American Journal of Cardiology, 55, 635-638.
Moritz	39	Theodorakis, Y. (1995). Effects of self-efficacy, satisfaction and personal goals on swimming performance. The SportPsychologist, 9,245-253.
Moritz	40	Treasure, D. C., Monson, J., & lox, C. l. (1996). Relationship between self-efficacy, wrestling performance, and affect priorto competition. The SportPsychologist, 10, 73-83.
Moritz	41	Vealey, R. (1986). Conceptualization of sport-confidence and competitive orientation: Preliminary investigation and instrument development. Journalof SportPsychology, 8, 221-246.
Moritz		Vealey sample 2
Moritz	42	Weinberg, R. S., Yukelson, D., & Jackson, A. (1980). Effects of public and private efficacyexpectations on competitive performance. Journal of SportPsychology, 2,340-349.
Moritz	43	Wells, C. M., Collins, D., & Hale, B. D. (1993). The self-efficacy performance link in maximum strength performance. Journalof SportSciences, tt, 167-175.
Moritz	44	Wilkes, R. t, &Summers, J. J. (1984). Cognitions, mediating variables, and strength performance. Journalof SportPsychology, 6,351-359.
Moritz	45	Woolfolk, R. t, Murphy, S.M., Gottesfeld, D., &Aitken, D. (1985). Effects of mental rehearsal of task motor activityand mental depiction of task outcome on motor skill performance. Journalof SportPsychology, 7, 191-197.
Search.One	1	Besharat, M. A., & Pourbohlool, S. (2011). Moderating effects of self-confidence and sport self-efficacy on the relationship between competitive anxiety and sport performance. Psychology, 2(7), 760–765. https://doi-org.lib-e2.lib.ttu.edu/10.4236/psych.2011.27116
Search.One	2	Arruza, J. A., Telletxea, S., De Montes, L. G., Arribas, S., Balagué, G., Cecchini, J. A., & Brustad, R. J. (2009). Understanding the relationship between perceived development of the competition plan and sport performance: Mediating effects of self-efficacy and state depression. Perceptual and Motor Skills, 109(1), 304–314. https://doi-org.lib-e2.lib.ttu.edu/10.2466/pms.109.1.304-314
Search.One	3	Miller, M., Carlyle, S., & Pease, R. (1992). The relationship between motivation and self-efficacy in competitive athletes participating in swimming, ice hockey, and basketball. Journal of Sport Behavior, 15(3), 201–208.

Search.One	4	Miller, M. (1993). Efficacy strength and performance in competitive swimmers of different skill levels. International Journal of Sport Psychology, 24(3), 284–296.
Search.One	5	Vilar, L., Araújo, D., Davids, K., & Renshaw, I. (2012). The need for "representative task design" in evaluating efficacy of skills tests in sport: A comment on Russell, Benton and Kingsley (2010). Journal of Sports Sciences, 30(16), 1727–1730. https://doi-org.lib-e2.lib.ttu.edu/10.1080/02640414.2012.679674
Search.One	6	Baretta, D., Greco, A., & Steca, P. (2017). Understanding performance in risky sport: The role of self-efficacy beliefs and sensation seeking in competitive freediving. Personality and Individual Differences, 117, 161–165. https://doi-org.lib-e2.lib.ttu.edu/10.1016/j.paid.2017.06.006
Search.One	7	Sklett, V. H., Lorås, H. W., & Sigmundsson, H. (2018). Self-efficacy, flow, affect, worry and performance in elite World Cup ski jumping. Frontiers in Psychology, 9. https://doi-org.lib-e2.lib.ttu.edu/10.3389/fpsyg.2018.01215
Search.One	8	Nicholls, A. R., Polman, R., & Levy, A. R. (2010). Coping self-efficacy, pre-competitive anxiety, and subjective performance among athletes. European Journal of Sport Science, 10(2), 97–102. https://doi-org.lib-e2.lib.ttu.edu/10.1080/17461390903271592
Search.One	9	Kuczka, K. K., & Treasure, D. C. (2005). Self-handicapping in competitive sport: Influence of the motivational climate, self-efficacy, and perceived importance. Psychology of Sport and Exercise, 6(5), 539–550. https://doi-org.lib-e2.lib.ttu.edu/10.1016/j.psychsport.2004.03.007
Search.One	10	Martin, J. J. (1995). Competitive orientation, self-efficacy and goal importance in Filipino marathoners. International Journal of Sport Psychology, 26(3), 348–358.
Search.One	11	Vealey, R. S. (2019). A periodization approach to building confidence in athletes. Journal of Sport Psychology in Action, 10(1), 26–37. https://doi-org.lib-e2.lib.ttu.edu/10.1080/21520704.2018.1496213
Search.One	12	Leonardi, T. J., Starapoli Martins, M. C., de Barros Gonçalves, C. E., Rodrigues Paes, R., & Gonçalves Moreira de Carvalho, H. J. (2019). Changes in tactical performance and self-efficacy on young female basketball players. / Variações na performance tática e na autoeficácia de jovens meninas atletas de basquetebol. Brazilian Journal of Kineanthropometry & Human Performance, 21, 1–9.
Search.One	13	Boat, R., & Taylor, I. M. (2015). Patterns of Change in Psychological Variables Leading up to Competition in Superior Versus Inferior Performers. Journal of Sport & Exercise Psychology, 37(3), 244–256.

Search.One	14	Bruton, A. M., Mellalieu, S. D., Shearer, D., Roderique-Davies, G., & Hall, R. (2013). Performance accomplishment information as predictors of self-efficacy as a function of skill level in amateur golf. Journal of Applied Sport Psychology, 25(2), 197–208. https://doi-org.lib-e2.lib.ttu.edu/10.1080/10413200.2012.705802
Search.One	15	Karimian, M., Kashefolhagh, F., Dadashi, M. S., & Chharbaghi, Z. (2010). The effect of relaxation and mental imagery on self-efficacy, competitive anxiety and sportive performance. British Journal of Sports Medicine, 44(S1), i57.
Search.One	16	Hazell, J., Cotterill, S. T., & Hill, D. M. (2014). An exploration of pre-performance routines, self-efficacy, anxiety and performance in semi-professional soccer. European Journal of Sport Science, 14(6), 603–610. https://doi-org.lib-e2.lib.ttu.edu/10.1080/17461391.2014.888484
Search.One	17	Pattinson, E. M., Cotterill, S. T., & Leyland, S. D. (2017). Sources of self-efficacy in springboard and highboard diving: A qualitative investigation. Sport & Exercise Psychology Review, 13(1), 80–91.
Search.One	18	García-Naveira Vaamonde, A. (2018). Optimismo, autoeficacia general y competitividad en jóvenes atletas de alto rendimiento. / Optimism, general self-efficacy and competitiveness in young high-performance athletes. Cultura, Ciencia y Deporte, 13(37), 71–81.
Search.One	19	Chase, M. A., Magyar, T. M., & Drake, B. M. (2005). Fear of injury in gymnastics: Self-efficacy and psychological strategies to keep on tumbling. Journal of Sports Sciences, 23(5), 465–475. https://doi-org.lib-e2.lib.ttu.edu/10.1080/02640410400021427
Search.One	20	Avugos, S., Bar-Eli, M., Ritov, I., & Sher, E. (2013). The elusive reality of efficacy–performance cycles in basketball shooting: An analysis of players' performance under invariant conditions. International Journal of Sport and Exercise Psychology, 11(2), 184–202. https://doi-org.lib-e2.lib.ttu.edu/10.1080/1612197X.2013.773661
Search.One	21	Head Over Heels With Success: The Relationship Between Self-Efficacy and Performance in Competitive Youth Gymnastics. (1989). Journal of Sport & Exercise Psychology, 11(4), 444–451.
Search.One	22	Liu, W., & Chepyator-Thomson, J. R. (2009). Field Dependence-Independence and Physical Activity Engagement among Middle School Students. Physical Education and Sport Pedagogy, 14(2), 125–136.
Search.One	23	Ramsey, R., Cumming, J., Edwards, M. G., Williams, S., & Brunning, C. (2010). Examining the emotion aspect of

		PETTLEP-based imagery with penalty taking in soccer. Journal of Sport Behavior, 33(3), 295–314.
Search.One	24	Bueno, J., Capdevila, L., & Fernández-Castro, J. (2002). Sufrimiento competitivo y rendimiento en deportes de resistencia = Competitive suffering and performance in endurance sports. Revista de Psicología Del Deporte, 11(2), 209–226.
Search.One	25	Smrdu, M. (2015). First-Person Experience of Optimal Sport Competition Performance of Elite Team Athletes. Kinesiology, 47(2), 169–178.
Search.One	26	Lee, C. (1982). Self-efficacy as a Predictor of Performance in Competitive Gymnastics. Journal of Sport Psychology, 4(4), 405–409.
Search.One	27	Psychountaki, M., & Zervas, Y. (2000). Competitive worries, sport confidence, and performance ratings for young swimmers. Perceptual and Motor Skills, 91(1), 87–94. https://doi-org.lib-e2.lib.ttu.edu/10.2466/PMS.91.5.87-94
Search.One	28	Scanlan, T. K., & Passer, M. W. (1979). Factors Influencing the Competitive Performance Expectancies of Young Female Athletes. Journal of Sport Psychology, 1(3), 212–220.
Search.One	29	Zabala, M., Morente-Sánchez, J., Mateo-March, M., & Sanabria, D. (2016). Relationship Between Self-Reported Doping Behavior and Psychosocial Factors in Adult Amateur Cyclists. Sport Psychologist, 30(1), 68–75.
Search.One	30	Hall, H. K., & Kerr, A. W. (1998). Predicting achievement anxiety: A social-cognitive perspective. Journal of Sport & Exercise Psychology, 20(1), 98–111.
Search.One	31	Slimani, M., Chamari, K., Boudhiba, D., & Chéour, F. (2016). Mediator and moderator variables of imagery use-motor learning and sport performance relationships: a narrative review. Sport Sciences for Health, 12(1), 1–9.
Search.One	32	Rojas, N. G. (2007). Implicaciones de la autoeficacia en el rendimiento deportivo = Implications of self-efficacy in sporting performance. Pensamiento Psicológico, 3(9), 21–32.
Search.One	33	Beaven, C. M. (2017). Mental Rehearsal Impacts Hormones and Subsequent Sport-Specific Performance. New Zealand Journal of Sports Medicine, 44(1), 41–42.
Search.One	34	Bruton, A. M., Shearer, D. A., & Mellalieu, S. D. (2019). Who said “there is no ‘I’ in team”? The effects of observational learning content level on efficacy beliefs in groups. Psychology of Sport & Exercise, 45, N.PAG.
Search.One	35	Meijen, C., Jones, M. V., McCarthy, P. J., Sheffield, D., & Allen, M. S. (2013). Cognitive and affective components of challenge and threat states. Journal of Sports Sciences, 31(8), 847–855. https://doi-org.lib-e2.lib.ttu.edu/10.1080/02640414.2012.753157

Search.One	36	Efficacy Expectations and Perceptions of Causality in Motor Performance. (1987). Journal of Sport Psychology, 9(4), 385–393.
Search.One	37	Otten, M. (2009). Choking vs clutch performance: A study of sport performance under pressure. Journal of Sport & Exercise Psychology, 31(5), 583–601. https://doi-org.lib-e2.lib.ttu.edu/10.1123/jsep.31.5.583
Search.One	38	Chow, G. M., Hepler, T. J., & Feltz, D. L. (2007). The effects of group motivational processes on individual performance in women's volleyball. Journal of Sport & Exercise Psychology, 29, S152–S153.
Search.One	39	Jones, M., Meijen, C., McCarthy, P. J., & Sheffield, D. (2009). A Theory of Challenge and Threat States in Athletes. International Review of Sport & Exercise Psychology, 2(2), 161–180.
Search.One	40	Middlemas, S., & Harwood, C. (2020). A pre-match video self-modeling intervention in elite youth football. Journal of Applied Sport Psychology, 32(5), 450–475. https://doi-org.lib-e2.lib.ttu.edu/10.1080/10413200.2019.1590481
Search.One	41	Boes, R., Harung, H. S., Travis, F., & Pensgaard, A. M. (2014). Mental and physical attributes defining world-class Norwegian athletes: Content analysis of interviews. Scandinavian Journal of Medicine & Science in Sports, 24(2), 422–427. https://doi-org.lib-e2.lib.ttu.edu/10.1111/j.1600-0838.2012.01498.x
Search.One	42	Thelwell, R. C., & Maynard, I. W. (2000). Professional cricketers' perceptions of the importance of antecedents influencing repeatable good performance. Perceptual and Motor Skills, 90(2), 649–658. https://doi-org.lib-e2.lib.ttu.edu/10.2466/PMS.90.2.649-658
Search.One	43	Turner, M. J., Jones, M. V., Sheffield, D., & Cross, S. L. (2012). Cardiovascular indices of challenge and threat states predict competitive performance. International Journal of Psychophysiology, 86(1), 48–57. https://doi-org.lib-e2.lib.ttu.edu/10.1016/j.ijpsycho.2012.08.004
Search.One	44	Fazlagić, A., & Belić, M. (2011). Lokus Kontrole I Flow Uspješnih Sportista. / Locus of Control and Flow of Successful Sportsman. Sportekspert, 4(1/2), 7–11.
Search.One	45	Deeter, T. E. (1990). Re-modeling expectancy and value in physical activity. Journal of Sport & Exercise Psychology, 12(1), 86–91.
Search.One	46	Gil Martínez, J., Capafons Bonet, A., & Labrador Encinas, F. (1993). Variables físicas y psicológicas predictoras del rendimiento deportivo y del cambio terapeútico = Physical and psychological variables predictive of sports output and therapeutical change. Psicothema, 5(1), 97–110.

Search.One	47	Suay, F., Salvador, A., González-Bono, E., Sanchís, C., Martínez, M., Martinéz-Sanchis, S., Simón, V. M., & Montoro, J. B. (1999). Effects of competition and its outcome on serum testosterone, cortisol and prolactin. Psychoneuroendocrinology, 24(5), 551–566. https://doi-org.lib-e2.lib.ttu.edu/10.1016/S0306-4530(99)00011-6
Search.One	48	Gernigon, C., Briki, W., & Eykens, K. (2010). The dynamics of psychological momentum in sport: The role of ongoing history of performance patterns. Journal of Sport & Exercise Psychology, 32(3), 377–400.
Search.One	49	Toering, T. T., Elferink-Gemser, M. T., Jordet, G., & Visscher, C. (2009). Self-regulation and performance level of elite and non-elite youth soccer players. Journal of Sports Sciences, 27(14), 1509–1517. https://doi-org.lib-e2.lib.ttu.edu/10.1080/02640410903369919
Search.One	50	WILLIAMS, E. L., JONES, H. S., SPARKS, S. A., MIDGLEY, A. W., MARCHANT, D. C., BRIDGE, C. A., & MCNAUGHTON, L. R. (2015). Altered Psychological Responses to Different Magnitudes of Deception during Cycling. Medicine & Science in Sports & Exercise, 47(11), 2423–2430.
Search.One	51	Greenlees, I. A., Nunn, R. L., Graydon, J. K., & Maynard, I. W. (1999). The relationship between collective efficacy and precompetitive affect in rugby players: Testing Bandura's model of collective efficacy. Perceptual and Motor Skills, 89(2), 431–440. https://doi-org.lib-e2.lib.ttu.edu/10.2466/PMS.89.6.431-440
Search.One	52	Donovan, J. J., & Williams, K. J. (2003). Missing the mark: Effects of time and causal attributions on goal revision in response to goal-performance discrepancies. Journal of Applied Psychology, 88(3), 379–390. https://doi-org.lib-e2.lib.ttu.edu/10.1037/0021-9010.88.3.379
Search.One	53	Wiggins, M. S. (1998). Anxiety intensity and direction: Preperformance temporal patterns and expectations in athletes. Journal of Applied Sport Psychology, 10(2), 201–211. https://doi-org.lib-e2.lib.ttu.edu/10.1080/10413209808406388
Search.One	54	Sheard, M., & Golby, J. (2006). Effect of a Psychological Skills Training Program on Swimming Performance and Positive Psychological Development. International Journal of Sport and Exercise Psychology, 4(2), 149–169. https://doi-org.lib-e2.lib.ttu.edu/10.1080/1612197X.2006.9671790
Search.One	55	Kingston, K. M., & Hardy, L. (1997). Effects of different types of goals on processes that support performance. The Sport Psychologist, 11(3), 277–293.
Search.One	56	Reifsteck, E. J., Anderson, S. N., Newton, J. D., & Maher, J. P. (2021). A practical guide and empirical example for

		implementing ecological momentary assessment in sport psychology research with athletes. Sport, Exercise, and Performance Psychology, 10(3), 408–422. https://doi-org.lib-e2.lib.ttu.edu/10.1037/spy0000252
Search.One	57	Coutts, A. J., & Reaburn, P. (2008). Monitoring changes in rugby league players' perceived stress and recovery during intensified training. Perceptual and Motor Skills, 106(3), 904–916. https://doi-org.lib-e2.lib.ttu.edu/10.2466/PMS.106.3.904-916
Search.One	58	Hall, C. R., Munroe-Chandler, K. J., Fishburne, G. J., & Hall, N. D. (2009). The Sport Imagery Questionnaire for Children (SIQ-C). Measurement in Physical Education and Exercise Science, 13(2), 93–107. https://doi-org.lib-e2.lib.ttu.edu/10.1080/10913670902812713
Search.One	59	McAuley, E., & Gill, D. (1983). Reliability and validity of the physical self-efficacy scale in a competitive sport setting. Journal of Sport Psychology, 5(4), 410–418.
Search.One	60	Martin, J. J., & Gill, D. L. (1995). The relationships of competitive orientations and self-efficacy to goal importance, thoughts, and performance in high school distance runners. Journal of Applied Sport Psychology, 7(1), 50–62. https://doi-org.lib-e2.lib.ttu.edu/10.1080/10413209508406300
Search.One	61	LaGuardia, R., & Labbé, E. E. (1993). Self-efficacy and anxiety and their relationship to training and race performance. Perceptual and Motor Skills, 77(1), 27–34. https://doi-org.lib-e2.lib.ttu.edu/10.2466/pms.1993.77.1.27
Search.One	62	George, T. R. (1994). Self-confidence and baseball performance: A causal examination of self-efficacy theory. Journal of Sport & Exercise Psychology, 16(4), 381–399.
Search.One	63	Kane, T. D., Marks, M. A., Zaccaro, S. J., & Blair, V. (1996). Self-efficacy, personal goals, and wrestlers' self-regulation. Journal of Sport & Exercise Psychology, 18(1), 36–48.
Search.Two	1	Gabriel, E. H., McCann, R. S., Hoch, M. C., & Cramer, R. J. (2020). An intervention based on the health belief model led to improvements in self-efficacy towards ERIPP participation and functional performance in club sport athletes: a pilot study. Journal of Science & Medicine in Sport, 23(10), 921–926.
Search.Two	2	Sivrikaya, M. H. (2018). The Role of Self-Efficacy on Performance of Sports Skills of Football Players. Journal of Education and Training Studies, 6, 75–79.
Search.Two	3	Coudevylle, G. R., Ginis, K. A. M., & Famose, J.-P. (2008). Determinants of self-handicapping strategies in sport and their effects on athletic performance. Social Behavior and

		Personality: An International Journal, 36(3), 391–398. https://doi-org.lib-e2.lib.ttu.edu/10.2224/sbp.2008.36.3.391
Search.Two	4	Wilhelm, A., Büsch, D., & Pabst, J. (2013). Sportspielspezifische Wirksamkeitserwartungen im Nachwuchsleistungshandball = Sport-game specific self-efficacy beliefs in handball. Zeitschrift Für Sportpsychologie, 20(4), 137–149. https://doi-org.lib-e2.lib.ttu.edu/10.1026/1612-5010/a000104
Search.Two	5	Ortiz Romero, M. T., Garrido Guzmán, M. E., & Castañeda Vázquez, C. (2022). Autoeficacia y resiliencia: diferencias entre deportistas practicantes de fitness/culturismo y no deportistas. / Self-efficacy and resilience: differences between fitness/bodybuilding athletes and nonathletes. Retos: Nuevas Perspectivas de Educación Física, Deporte y Recreación, 44, 232–241.
Search.Two	6	Stonecypher, J. M., Blom, L. C., Johnson, J. E., Bolin, J. H., & Hilliard, R. C. (2019). Interdependent tripartite efficacy perceptions and individual performance: Case study of a boys' basketball team. Psychological Reports, 122(2), 645–669. https://doi-org.lib-e2.lib.ttu.edu/10.1177/0033294118761045
Search.Two	7	Sklett, V. H., Lorås, H. W., & Sigmundsson, H. (2018). Self-efficacy, flow, affect, worry and performance in elite World Cup ski jumping. Frontiers in Psychology, 9. https://doi-org.lib-e2.lib.ttu.edu/10.3389/fpsyg.2018.01215
Search.Two	8	Molina, V. M., Oriol, X., & Mendoza, M. C. (2018). Emotional regulation and physical recovery in young athletes of individual and collective sport modalities. / Regulación emocional y recuperación física de los jóvenes deportistas en modalidades deportivas individual y colectiva. RICYDE. Revista Internacional de Ciencias Del Deporte, 14(53), 191–204.
Search.Two	9	Hepler, T. J., & Chase, M. A. (2008). Relationship between decision-making self-efficacy, task self-efficacy, and the performance of a sport skill. Journal of Sports Sciences, 26(6), 603–610. https://doi-org.lib-e2.lib.ttu.edu/10.1080/02640410701654280
Search.Two	10	Sallen, J., Hemming, K., & Richartz, A. (2018). Facilitating dual careers by improving resistance to chronic stress: effects of an intervention programme for elite student athletes. European Journal of Sport Science, 18(1), 112–122.
Search.Two	11	PIERPAOLO, L., & ANTONIA, T. G. (2018). The psychological constructs and dimensions applied to sports performance: a change of theoretical paradigms. Journal of Physical Education & Sport, 18, 2034–2038.
Search.Two	12	LaForge-MacKenzie, K., & Sullivan, P. J. (2014). The relationship between self-efficacy and performance within a

		continuous educational gymnastics routine. International Journal of Sport and Exercise Psychology, 12(3), 206–217. https://doi-org.lib-e2.lib.ttu.edu/10.1080/1612197X.2014.909511
Search.Two	13	Teques, P., Calmeiro, L., Rosado, A., Silva, C., & Serpa, S. (2019). Perceptions of parenting practices and psychological variables of elite and sub-elite youth athletes. Frontiers in Psychology, 10. https://doi-org.lib-e2.lib.ttu.edu/10.3389/fpsyg.2019.01495
Search.Two	14	Kolovelonis, A. (2019). Relating Students' Participation in Sport out of School and Performance Calibration in Physical Education. Issues in Educational Research, 29(3), 774–789.
Search.Two	15	Hutchinson, J. C., Sherman, T., Martinovic, N., & Tenenbaum, G. (2008). The effect of manipulated self-efficacy on perceived and sustained effort. Journal of Applied Sport Psychology, 20(4), 457–472. https://doi-org.lib-e2.lib.ttu.edu/10.1080/10413200802351151
Search.Two	16	Turner, M. J., Jones, M. V., Sheffield, D., Slater, M. J., Barker, J. B., & Bell, J. J. (2013). Who thrives under pressure? Predicting the performance of elite academy cricketers using the cardiovascular indicators of challenge and threat states. Journal of Sport & Exercise Psychology, 35(4), 387–397. https://doi-org.lib-e2.lib.ttu.edu/10.1123/jsep.35.4.387
Search.Two	17	Rouquette, O. Y., Knight, C. J., Lovett, V. E., & Heuzé, J.-P. (2021). The influence of perceived parental responsiveness on athletes' goal accomplishment, trait cognitive sport anxiety, and thriving: A semi-longitudinal study. Psychology of Sport & Exercise, 57, N.PAG.
Search.Two	18	Haney, C. J., & Long, B. C. (1995). Coping effectiveness: a path analysis of self-efficacy, control, coping, and performance in sport competitions. Journal of Applied Social Psychology, 25, 1726–1746.
Search.Two	19	Perkins, N. (2018). Positive Impact of Participation in Wheelchair Rugby: A Review of the Literature. Palaestra, 32(2), 18–22.
Search.Two	20	Watkins, B., Garcia, A. W., & Turek, E. (1994). The relation between self-efficacy and sport performance: evidence from a sample of youth baseball players. / La relation entre l ' efficacite et la performance sportive - analyse a partir d ' un echantillon de jeunes joueurs de baseball. Journal of Applied Sport Psychology, 6(1), 21–31.
Search.Two	21	Sarrazin, P., & Famose, J.-P. (2005). Plus c'est difficile et mieux je réussis! Étude des liens entre les buts fixés, l'efficacité personnelle et la performance sportive = It is difficult and I am afraid to fail! Study of the relation among fixed goals, personal efficacy, and sports performance.

		Bulletin de Psychologie, 58(1), 107–111. https://doi-org.lib-e2.lib.ttu.edu/10.3917/bupsy.475.0107
Search.Two	22	RINTAUGU, E. G., MWANGI, F. M., & TORIOLA, A. L. (2018). Sources of sports confidence and contextual factors among university athletes. Journal of Physical Education & Sport, 18(2), 889–895.
Search.Two	23	Barker, J. B., Jones, M. V., & Greenlees, I. (2013). Using hypnosis to enhance self-efficacy in sport performers. Journal of Clinical Sport Psychology, 7(3), 228–247. https://doi-org.lib-e2.lib.ttu.edu/10.1123/jcsp.7.3.228
Search.Two	24	Pettit, J. A., & Karageorghis, C. I. (2020). Effects of video, priming, and music on motivation and self-efficacy in American football players. International Journal of Sports Science & Coaching, 15(5/6), 685–695.
Search.Two	25	Slobounov, S., Yukelson, D., & O'Brien, R. (1997). Self-efficacy and movement variability of Olympic-level springboard divers. Journal of Applied Sport Psychology, 9(2), 171–190. https://doi-org.lib-e2.lib.ttu.edu/10.1080/10413209708406480
Search.Two	26	Winfrey, M. L., & Weeks, D. L. (1993). Effects of self-modeling on self-efficacy and balance beam performance. Perceptual and Motor Skills, 77(3, Pt 1), 907–913. https://doi-org.lib-e2.lib.ttu.edu/10.2466/pms.1993.77.3.907
Search.Two	27	Gouvêa, F. C. (2003). Análise Da Auto-Eficácia Em Atletas De Modalidades Individuais E Coletivas. / Analysis of the Self-Efficacy in Athletes of Individual and Collective Modalities. Revista Mackenzie de Educacao Fisica e Esporte, 2(2), 45–59.
Search.Two	28	Horcajo J, Santos D, Higuero G. The effects of self-efficacy on physical and cognitive performance: An analysis of meta-certainty. Psychology of Sport and Exercise. 2022;58. doi:10.1016/j.psychsport.2021.102063
Search.Two	29	Law, B., & Ste-Marie, D. M. (2005). Effects of self-modeling on figure skating jump performance and psychological variables. European Journal of Sport Science, 5(3), 143–152. https://doi-org.lib-e2.lib.ttu.edu/10.1080/17461390500159273
Search.Two	30	Irazusta, S., & Arruza, J. (2006). Influencia de Variables Psicológicas en el Rendimiento de Jugadores Amateurs de Golf = The Influence of Psychological Variables on Amateur Golfers' Performance. Revista de Psicología Del Deporte, 15(1), 127–138.
Search.Two	31	Iaochite, R. T., & Vieira, R. R. (2013). Autoeficácia De Técnicos Esportivos: Um Estudo Exploratório1. / Coach Self-Efficacy: An Exploratory Study. Revista Mackenzie de Educacao Fisica e Esporte, 12(1), 79–94.
Search.Two	32	Blecharz, J., Luszczynska, A., Tenenbaum, G., Scholz, U., & Cieslak, R. (2014). Self-efficacy moderates but collective

		efficacy mediates between motivational climate and athletes' well-being. Applied Psychology: Health and Well-Being, 6(3), 280–299. https://doi-org.lib-e2.lib.ttu.edu/10.1111/aphw.12028
Search.Two	33	Aldous-Granby, J., & McCormick, A. (2020). Effects of clothing perception on psychological factors and tactical intentions in fencing. International Journal of Sport & Exercise Psychology, 18(3), 380–390.
Search.Two	34	Lane, A. M., Hall, R., & Lane, J. (2004). Self-Efficacy and Statistics Performance among Sport Studies Students. Teaching in Higher Education, 9(4), 435–448.
Search.Two	35	Stidwill, H. F. (1994). Application of self-efficacy theory: A treatment approach for sport performance phobias. Journal of Mental Health Counseling, 16(2), 196–204.
Search.Two	36	González, J., & Garcés de los Fayos, E. J. (2014). Diferencias indfvtduales e indicadores de ansiedad como estrategias adaptativas de autoeficacia en el deportista = Individual differences and indicators of anxiety as adaptive strategies of self-efficacy in athletes. Ansiedad y Estrés, 20(1), 51–59.
Search.Two	37	Saville, P. D., Bray, S. R., Ginis, K. A. M., Cairney, J., Marinoff-Shupe, D., & Pettit, A. (2014). Sources of self-efficacy and coach/instructor behaviors underlying relation-inferred self-efficacy (RISE) in recreational youth sport. Journal of Sport & Exercise Psychology, 36(2), 146–156. https://doi-org.lib-e2.lib.ttu.edu/10.1123/jsep.2013-0144
Search.Two	38	Blecharz, J., Horodyska, K., Zarychta, K., Adamiec, A., & Luszczynska, A. (2015). Intrinsic motivation predicting performance satisfaction in athletes: Further psychometric evaluations of the Sport Motivation Scale-6. Polish Psychological Bulletin, 46(2), 309–319.
Search.Two	39	ESTEVAN, I., ÁLVAREZ, O., FALCÓ, C., & CASTILLO, I. (2014). Self-efficacy and performance of the roundhouse kick in taekwondo. / Autoeficacia y rendimiento en la patada circular de taekwondo. Revista de Artes Marciales Asiaticas, 9(2), 97–105.
Search.Two	40	Samson, A. (2014). Sources of self-efficacy during marathon training: A qualitative, longitudinal investigation. The Sport Psychologist, 28(2), 164–175. https://doi-org.lib-e2.lib.ttu.edu/10.1123/tsp.2013-0027
Search.Two	41	HEAZLEWOOD, I., & BURKE, S. (2011). Self-efficacy and its relationship to selected sport psychological constructs in the prediction of performance in ironman triathlon. Journal of Human Sport & Exercise, 6(2), 328–350.
Search.Two	42	Yang, Z. (2020). An empirical analysis of the relationship between self-efficacy, motivation, and team performance of high-level basketball players. Revista de Psicología Del Deporte, 29(4), 221–231.

Search.Two	43	Turner, M. J., Massie, R., Slater, M. J., & Braithwaite, E. (2021). Do challenge and threat evaluations predict netball performance and selection at trials in youth netball players? Sport, Exercise, and Performance Psychology, 10(1), 71–87. https://doi-org.lib-e2.lib.ttu.edu/10.1037/spy0000248
Search.Two	44	Fiander, M. F., Jones, M. I., & Parker, J. K. (2018). An exploration of game-strategy efficacy beliefs in UK youth sport coaches. Sport & Exercise Psychology Review, 14(1), 33–46.
Search.Two	45	Jonker, L., Elferink-Gemser, M., Toering, T., Lyons, J., & Visscher, C. (2010). Academic performance and self-regulatory skills in elite youth soccer players. Journal of Sports Sciences, 28(14), 1605–1614.
Search.Two	46	Beattie, S., Lief, D., Adamoulas, M., & Oliver, E. (2011). Investigating the possible negative effects of self-efficacy upon golf putting performance. Psychology of Sport and Exercise, 12(4), 434–441. https://doi-org.lib-e2.lib.ttu.edu/10.1016/j.psychsport.2011.02.004
Search.Two	47	McCormick, A., Meijen, C., & Marcora, S. (2015). Psychological determinants of whole-body endurance performance. Sports Medicine, 45(7), 997–1015. https://doi-org.lib-e2.lib.ttu.edu/10.1007/s40279-015-0319-6
Search.Two	48	Estevan, I., Álvarez, O., & Castillo, I. (2016). Autoeficacia percibida y rendimiento técnico-táctico en taekwondistas universitarios = Perceived self-efficacy and technical-tactical performance in university taekwondo athletes. Cuadernos de Psicología Del Deporte, 16(2), 51–64.
Search.Two	49	Barker, J. B., & Jones, M. V. (2008). The effects of hypnosis on self-efficacy, affect, and soccer performance: A case study. Journal of Clinical Sport Psychology, 2(2), 127–147.
Search.Two	50	Stander, F. W., Rothmann, S., & Botha, E. (2015). The role of teammate relationships, communication and self-efficacy in predicting athlete flow experience. Journal of Psychology in Africa, 25(6), 494–503.
Search.Two	51	Habeeb, C. M., Eklund, R. C., & Coffee, P. (2017). It depends on the partner: Person-related sources of efficacy beliefs and performance for athlete pairs. Journal of Sport & Exercise Psychology, 39(3), 172–187. https://doi-org.lib-e2.lib.ttu.edu/10.1123/jsep.2016-0348
Search.Two	52	Weigand, D. A., & Stockham, K. J. (2000). The importance of analyzing position–specific self-efficacy. Journal of Sport Behavior, 23(1), 61–69.
Search.Two	53	Cohen, A. B., Tenenbaum, G., & English, R. W. (2006). Emotions and Golf Performance: An IZOF-Based Applied Sport Psychology Case Study. Behavior Modification, 30(3), 259–280. https://doi-org.lib-e2.lib.ttu.edu/10.1177/0145445503261174

Search.Two	54	Leirdal, S., Sandbakk, Ø., & Ettema, G. (2013). Effects of frequency on gross efficiency and performance in roller ski skating. Scandinavian Journal of Medicine & Science in Sports, 23(3), 295–302. https://doi-org.lib-e2.lib.ttu.edu/10.1111/j.1600-0838.2011.01379.x
Search.Two	55	May, K. H., Guccione, A. A., Edwards, M. C., & Goldstein, M. S. (2016). The Adolescent Measure of Confidence and Musculoskeletal Performance (Amcamp): Development and Initial Validation. International Journal of Sports Physical Therapy, 11(5), 698–707.
Search.Two	56	Martin, J. J. (2002). Training and performance self-efficacy, affect, and performance in wheelchair road racers. The Sport Psychologist, 16(4), 384–395.
Search.Two	57	Kingsley, M., Russell, M., & Benton, D. (2012). Authors' response to letter to the Editor: "The need for 'representative task design' in evaluating efficacy of skills tests in sport: A comment on Russell, Benton and Kingsley (2010)." Journal of Sports Sciences, 30(16), 1731–1733. https://doi-org.lib-e2.lib.ttu.edu/10.1080/02640414.2012.723134
Search.Two	58	Öncü, E., Feltz, D. L., Lirgg, C. D., & Gürbüz, B. (2018). The examination of the psychometric properties of the Turkish Collective Efficacy Questionnaire for Sports. Acta Gymnica, 48(1), 27–35.
Search.Two	59	Jackson, B., & Beauchamp, M. R. (2007). Self-efficacy and performance in athlete dyads: Evidence of actor and partner effects. Journal of Sport & Exercise Psychology, 29, S173.
Search.Two	60	Law, B., & Hall, C. (2009). Observational learning use and self-efficacy beliefs in adult sport novices. Psychology of Sport and Exercise, 10(2), 263–270. https://doi-org.lib-e2.lib.ttu.edu/10.1016/j.psychsport.2008.08.003
Search.Two	61	Morris, R. L., & Kavussanu, M. (2008). Antecedents of approach-avoidance goals in sport. Journal of Sports Sciences, 26(5), 465–476. https://doi-org.lib-e2.lib.ttu.edu/10.1080/02640410701579388
Search.Two	62	Dallmann, P., Bach, C., Zipser, H., Thomann, P. A., & Herpertz, S. C. (2016). Evaluation of a stress prevention program for young high-performance athletes. Mental Health and Prevention, 4(2), 75–80. https://doi-org.lib-e2.lib.ttu.edu/10.1016/j.mhp.2016.04.001
Search.Two	63	Hofseth, E., Toering, T., Jordet, G., & Ivarsson, A. (2017). Self-evaluation of skills and performance level in youth elite soccer: Are positive self-evaluations always positive? Sport, Exercise, and Performance Psychology, 6(4), 370–383. https://doi-org.lib-e2.lib.ttu.edu/10.1037/spy0000094

Search.Two	64	Ram, N., & McCullagh, P. (2003). Self-modeling: Influence on psychological responses and physical performance. The Sport Psychologist, 17(2), 220–241.
Search.Two	65	Vasconcelos-Raposo, J. J. B., & Carvalho, R. (2000). Ansiedad cognitiva, somática y autoconfianza en árbitros de balonvolea = Cognitive and somatic anxiety, and self-efficacy in volleyball officials. Revista de Psicología Del Deporte, 9(1–2), 135–142.
Search.Two	66	Beauchamp, M. R., Bray, S. R., & Albinson, J. G. (2002). Pre-competition imagery, self-efficacy and performance in collegiate golfers. Journal of Sports Sciences, 20(9), 697–705.
Search.Two	67	Bortoli, L., Bertollo, M., Comani, S., & Robazza, C. (2011). Competence, achievement goals, motivational climate, and pleasant psychobiosocial states in youth sport. Journal of Sports Sciences, 29(2), 171–180. https://doi-org.lib-e2.lib.ttu.edu/10.1080/02640414.2010.530675
Search.Two	68	Miles, A., & Neil, R. (2013). The use of self-talk during elite cricket batting performance. Psychology of Sport and Exercise, 14(6), 874–881. https://doi-org.lib-e2.lib.ttu.edu/10.1016/j.psychsport.2013.07.005
Search.Two	69	Otter, R. T. A., Brink, M. S., van der Does, H. T. D., & Lemmink, K. A. P. M. (2016). Monitoring Perceived Stress and Recovery in Relation to Cycling Performance in Female Athletes. International Journal of Sports Medicine, 37(1), 12–18.
Search.Two	70	Wurtele, S. K. (1986). Self-efficacy and athletic performance: A review. Journal of Social and Clinical Psychology, 4(3), 290–301. https://doi-org.lib-e2.lib.ttu.edu/10.1521/jscp.1986.4.3.290
Search.Two	71	Ramires, A., Carapeta, C., Felgueiras, F., & Viana, M. F. (2001). Treino de modelagem e visualização mental: Avaliação dos efeitos nas expectativas de auto-eficácia e desempenho de atletas de patinagem = Training in modeling and mental visualization: Evaluation of the effects of expectations on self-efficacy and performance of skating athletes. Análise Psicológica, 19(1), 15–25.
Search.Two	72	Fransen, K., Steffens, N. K., Haslam, S. A., Vanbeselaere, N., Vande Broek, G., & Boen, F. (2016). We will be champions: Leaders' confidence in 'us' inspires team members' team confidence and performance. Scandinavian Journal of Medicine & Science in Sports, 26(12), 1455–1469. https://doi-org.lib-e2.lib.ttu.edu/10.1111/sms.12603
Search.Two	73	Boardley, I. D., Kavussanu, M., & Ring, C. (2008). Athletes' perceptions of coaching effectiveness and athlete-related outcomes in rugby union: An investigation based on the coaching efficacy model. The Sport Psychologist, 22(3), 269–287.

Search.Two	74	Christensen, D. S., & Smith, R. E. (2016). Psychological coping skills as predictors of collegiate golf performance: Social desirability as a suppressor variable. Sport, Exercise, and Performance Psychology, 5(1), 67–80. https://doi-org.lib-e2.lib.ttu.edu/10.1037/spy0000049
Search.Two	75	*Cockerill, I. M., Pyle, H. J., & Read, S. (1996). The self-efficacy/self-esteem relationship in sport and at work. Employee Counselling Today, 8(4), 19–23. https://doi-org.lib-e2.lib.ttu.edu/10.1108/13665629610121632*
Search.Two	76	Coffee, P., & Rees, T. (2011). When the chips are down: Effects of attributional feedback on self-efficacy and task performance following initial and repeated failure. Journal of Sports Sciences, 29(3), 235–245. https://doi-org.lib-e2.lib.ttu.edu/10.1080/02640414.2010.531752
Search.Two	77	Keeler, L. A., & Watson, J. C., II. (2011). The transtheoretical model and psychological skills training: Application and implications with elite female athletes. Athletic Insight: The Online Journal of Sport Psychology, 3(2), 95–113.
Search.Two	78	Gilson, T. A., Chow, G. M., & Feltz, D. L. (2012). Self-efficacy and athletic squat performance: Positive or negative influences at the within- and between-levels of analysis. Journal of Applied Social Psychology, 42(6), 1467–1485. https://doi-org.lib-e2.lib.ttu.edu/10.1111/j.1559-1816.2012.00908.x
Search.Two	79	Brown, L. J., Malouff, J. M., & Schutte, N. S. (2005). The Effectiveness of a Self-Efficacy Intervention for Helping Adolescents Cope with Sport-Competition Loss. Journal of Sport Behavior, 28(2), 136–150.
Search.Two	80	Gernigon, C., & Delloye, J.-B. (2003). Self-efficacy, causal attribution, and track athletic performance following unexpected success or failure among elite sprinters. The Sport Psychologist, 17(1), 55–76.
Search.Two	81	Hatzigeorgiadis, A., Zourbanos, N., Goltsios, C., & Theodorakis, Y. (2008). Investigating the functions of self-talk: The effects of motivational self-talk on self-efficacy and performance in young tennis players. The Sport Psychologist, 22(4), 458–471.
Search.Two	82	Dodge, T., & Jaccard, J. J. (2008). Is abstinence an alternative? Predicting adolescent athletes' intentions to use performance enhancing substances. Journal of Health Psychology, 13(5), 703–711. https://doi-org.lib-e2.lib.ttu.edu/10.1177/1359105307082460
Search.Two	83	Lowther, J., Lane, A., & Lane, H. (2002). Self-efficacy and psychological skills during the Amputee Soccer World Cup. Athletic Insight: The Online Journal of Sport Psychology, 4(2).

Search.Two	84	Garza, D. L., & Feltz, D. L. (1998). Effects of selected mental practice on performance, self-efficacy, and competition confidence of figure skaters. The Sport Psychologist, 12(1), 1–15.
Search.Two	85	Geisler, G. W. W., & Leith, L. M. (1997). The effects of self-esteem, self-efficacy and audience presence on soccer penalty shot performance. Journal of Sport Behavior, 20(3), 322–337.
Search.Two	86	Blecharz, J., Luszczynska, A., Scholz, U., Schwarzer, R., Siekanska, M., & Cieslak, R. (2014). Predicting performance and performance satisfaction: Mindfulness and beliefs about the ability to deal with social barriers in sport. Anxiety, Stress & Coping: An International Journal, 27(3), 270–287. https://doi-org.lib-e2.lib.ttu.edu/10.1080/10615806.2013.839989
Search.Two	87	Arias, J. L. (2012). Influence of ball weight on shot accuracy and efficacy among 9-11-year-old male basketball players. Kinesiology, 44(1), 52–59.
Search.Two	88	Morton, P. A. (2003). The hypnotic belay in alpine mountaineering: The use of self-hypnosis for the resolution of sports injuries and for performance enhancement. American Journal of Clinical Hypnosis, 46(1), 45–51. https://doi-org.lib-e2.lib.ttu.edu/10.1080/00029157.2003.10403564
Search.Two	89	Barkoukis, V., Koidou, E., & Tsorbatzoudis, H. (2010). Effects of a motivational climate intervention on state anxiety, self-efficacy, and skill development in physical education. European Journal of Sport Science, 10(3), 167–177. https://doi-org.lib-e2.lib.ttu.edu/10.1080/17461390903426634
Search.Two	90	Feltz, D. L., & Lirgg, C. D. (1998). Perceived team and player efficacy in hockey. Journal of Applied Psychology, 83(4), 557–564. https://doi-org.lib-e2.lib.ttu.edu/10.1037/0021-9010.83.4.557
Search.Two	91	Lohasz, P. G., & Leith, L. M. (1997). The effect of three mental preparation strategies on the performance of a complex response time task. International Journal of Sport Psychology, 28(1), 25–34.
Search.Two	92	Kitsantas, A., & Zimmerman, B. J. (2002). Comparing self-regulatory processes among novice, non-expert, and expert volleyball players: A microanalytic study. Journal of Applied Sport Psychology, 14(2), 91–105. https://doi-org.lib-e2.lib.ttu.edu/10.1080/10413200252907761
Search.Two	93	Wilson, M., & Smith, N. C. (2007). A test of the predictions of processing efficiency theory during elite team competition using the Thought Occurrence Questionnaire

		for Sport. International Journal of Sport Psychology, 38(3), 245–262.
Search.Two	94	Kalasountas, V., Reed, J., & Fitzpatrick, J. (2007). The effect of placebo-induced changes in expectancies on maximal force production in college students. Journal of Applied Sport Psychology, 19(1), 116–124. https://doi-org.lib-e2.lib.ttu.edu/10.1080/10413200601123736
Search.Two	95	McCarthy, P. J. (2011). Positive emotion in sport performance: Current status and future directions. International Review of Sport and Exercise Psychology, 4(1), 50–69. https://doi-org.lib-e2.lib.ttu.edu/10.1080/1750984X.2011.560955
Search.Two	96	Edwards, C., Tod, D., & McGuigan, M. (2008). Self-talk influences vertical jump performance and kinematics in male rugby union players. Journal of Sports Sciences, 26(13), 1459–1465. https://doi-org.lib-e2.lib.ttu.edu/10.1080/02640410802287071
Search.Two	97	Myers, N. D., Payment, C. A., & Feltz, D. L. (2004). Reciprocal Relationships Between Collective Efficacy and Team Performance in Women's Ice Hockey. Group Dynamics: Theory, Research, and Practice, 8(3), 182–195. https://doi-org.lib-e2.lib.ttu.edu/10.1037/1089-2699.8.3.182
Search.Two	98	Theodorakis, Y., Weinberg, R., Natsis, P., Douma, I., & Kazakas, P. (2000). The effects of motivational versus instructional self-talk on improving motor performance. The Sport Psychologist, 14(3), 253–271.
Search.Two	99	Sánchez, X., & Torregrosa, M. (2005). El papel de los factores psicológicos en la escalada deportiva: Un análisis cualitativo = The influence of psychological factors on sport performance. Revista de Psicología Del Deporte, 14(2), 177–194.
Search.Two	100	Musculus, L., Raab, M., Belling, P., & Lobinger, B. (2018). Linking self-efficacy and decision-making processes in developing soccer players. Psychology of Sport & Exercise, 39, 72–80.
Search.Two	101	Martínez-Romero, M., Molina, V. M., & Oriol-Granado, X. (2016). Desarrollo y validación del cuestionario de autoconfianza en competición CACD para deportes individuales y colectivos = Development and validation of questionnaire CAD confidence in competition for individual and team sports. Cuadernos de Psicología Del Deporte, 16(2), 13–20.
Search.Two	102	Schinke, R. J., & da Costa, J. L. (2001). A plausible relationship between support-infrastructure and major-games athletic performance. Athletic Insight: The Online Journal of Sport Psychology, 3(2).

Search.Two	103	Moll, T., Rees, T., & Freeman, P. (2017). Enacted support and golf-putting performance: The role of support type and support visibility. Psychology of Sport and Exercise, 30, 30–37. https://doi-org.lib-e2.lib.ttu.edu/10.1016/j.psychsport.2017.01.007
Search.Two	104	SHERIDAN, A., MARCHANT, D. C., WILLIAMS, E. L., JONES, H. S., HEWITT, P. A., & SPARKS, A. (2019). Presence of Spotters Improves Bench Press Performance: A Deception Study. Journal of Strength & Conditioning Research (Lippincott Williams & Wilkins), 33(7), 1755–1761.
Search.Two	105	Adler, A. B., Bliese, P. D., Pickering, M. A., Hammermeister, J., Williams, J., Harada, C., Csoka, L., Holliday, B., & Ohlson, C. (2015). Mental skills training with basic combat training soldiers: A group-randomized trial. Journal of Applied Psychology, 100(6), 1752–1764. https://doi-org.lib-e2.lib.ttu.edu/10.1037/apl0000021
Search.Two	106	CORRION, K., GERNIGON, C., DEBOIS, N., & D'ARRIPE-LONGUEVILLE, F. (2013). Factor validity and Reliability of the Resistive Self-Regulatory Efficacy in Sport Scale (RSRESS) in a French sample. International Journal of Sport Psychology, 44(2), 128–144.
Search.Two	107	引原 有輝, 渡邊 將司, 川勝 佐希, & 石井 好二郎. (2018). 子どもにおける運動・スポーツ活動と運動遊びの意義とは ーヘルスアウトカムとの関連から探るー / Benefits of organized sports participation and voluntary outdoor play in children and adolescents based on evidence-related health outcomes. Japanese Journal of Physical Fitness & Sports Medicine, 67(1), 83–98.
Search.Two	108	Bono, B., & Livi, S. (2016). Motivazione al successo in atleti di élite: applicazione del 2X2 Achievement Goal Framework nel nuoto = Achievement motivation in elite athletes: Application of 2X2 Achievement Goal Framework in swimming. Rassegna Di Psicologia, 33(1), 51–66.
Search.Two	109	Spink, K. S. (1990). Collective efficacy in the sport setting. International Journal of Sport Psychology, 21(4), 380–395.
Search.Two	110	di Fronso, S., Nakamura, F. Y., Bortoli, L., Robazza, C., & Bertollo, M. (2013). Stress and Recovery Balance in Amateur Basketball Players: Differences by Gender and Preparation Phase. International Journal of Sports Physiology & Performance, 8(6), 618–622.
Search.Two	111	Sappington, R., & Longshore, K. (2015). Systematically reviewing the efficacy of mindfulness-based interventions for enhanced athletic performance. Journal of Clinical Sport Psychology, 9(3), 232–262. https://doi-org.lib-e2.lib.ttu.edu/10.1123/jcsp.2014-0017
Search.Two	112	LaForge-MacKenzie, K., & Sullivan, P. J. (2014). A comparison of the self-efficacy-performance relationship

		between continuous and noncontinuous sport conditions. Journal of Applied Sport Psychology, 26(3), 363–376. https://doi-org.lib-e2.lib.ttu.edu/10.1080/10413200.2014.894154
Search.Two	113	Knicker, A. J., Renshaw, I., Oldham, A. R. H., & Cairns, S. P. (2011). Interactive Processes Link the Multiple Symptoms of Fatigue in Sport Competition. Sports Medicine, 41(4), 307–328.
Search.Two	114	Sit, C. H. P., & Lindner, K. J. (2007). Achievement goal profiles, perceived ability and participation motivation for sport and physical activity. International Journal of Sport Psychology, 38(3), 283–303.
Search.Two	115	Feltz, D. L., & Weiss, M. R. (1982). Developing self-efficacy through sport. Journal of Physical Education, Recreation & Dance, 53(3), 24–26.
Search.Two	116	Marsh, H. W., Chanal, J. P., & Sarrazin, P. G. (2006). Self-belief does make a difference: A reciprocal effects model of the causal ordering of physical self-concept and gymnastics performance. Journal of Sports Sciences, 24(1), 101–111. https://doi-org.lib-e2.lib.ttu.edu/10.1080/02640410500130920
Search.Two	117	Skjesol, K., & Halvari, H. (2005). Motivational Climate, Achievement Goals, Perceived Sport Competence, and Involvement in Physical Activity: Structural and Mediator Models. Perceptual and Motor Skills, 100(2), 497–523. https://doi-org.lib-e2.lib.ttu.edu/10.2466/PMS.100.2.497-523
Search.Two	118	Gilbert, W., Dubina, N., & Emmett, M. (2012). "Exploring the potential of assessment efficacy in sports coaching": A commentary. International Journal of Sports Science & Coaching, 7(2), 211–214. https://doi-org.lib-e2.lib.ttu.edu/10.1260/1747-9541.7.2.211
Search.Two	119	Hay, P., Dickens, S., Crudgington, B., & Engstrom, C. (2012). Exploring the potential of assessment efficacy in sports coaching. International Journal of Sports Science & Coaching, 7(2), 187–198. https://doi-org.lib-e2.lib.ttu.edu/10.1260/1747-9541.7.2.187
Search.Two	120	Burke, S. T., & Jin, P. (1996). Predicting performance from a triathlon event. Journal of Sport Behavior, 19(4), 272–287.
Search.Two	121	Reade, I. (2012). "Exploring the potential of assessment efficacy in sports coaching": A commentary. International Journal of Sports Science & Coaching, 7(2), 199–202. https://doi-org.lib-e2.lib.ttu.edu/10.1260/1747-9541.7.2.199
Search.Two	122	KING, D., CLARK, T., KELLMANN, M., & HUME, P. A. (2016). Stress and recovery changes of injured and non-injured amateur representative rugby league players over a competition season. New Zealand Journal of Sports Medicine, 43(2), 57–63.

Search.Two	123	Hay, P., Dickens, S., Crudgington, B., & Engstrom, C. (2012). "Exploring the potential of assessment efficacy in sports coaching": A response to commentaries. International Journal of Sports Science & Coaching, 7(2), 223–226. https://doi-org.lib-e2.lib.ttu.edu/10.1260/1747-9541.7.2.223
Search.Two	124	*Bueno, J., Weinberg, R. S., Fernández-Castro, J., & Capdevila, L. (2008). Emotional and motivational mechanisms mediating the influence of goal setting on endurance athletes' performance. Psychology of Sport and Exercise, 9(6), 786–799. https://doi-org.lib-e2.lib.ttu.edu/10.1016/j.psychsport.2007.11.003*
Search.Two	125	Pineau, T. R., Glass, C. R., Kaufman, K. A., & Bernal, D. R. (2014). Self- and team-efficacy beliefs of rowers and their relation to mindfulness and flow. Journal of Clinical Sport Psychology, 8(2), 142–158. https://doi-org.lib-e2.lib.ttu.edu/10.1123/jcsp.2014-0019
Search.Two	126	Fry, M. D., Guivernau, M., Kim, M., Newton, M., Gano-Overway, L. A., & Magyar, T. M. (2012). Youth perceptions of a caring climate, emotional regulation, and psychological well-being. Sport, Exercise, and Performance Psychology, 1(1), 44–57. https://doi-org.lib-e2.lib.ttu.edu/10.1037/a0025454
Search.Two	127	Gonzalez-Bono, E., Salvador, A., Ricarte, J., Serrano, M. A., & Arnedo, M. (2000). Testosterone and attribution of successful competition. Aggressive Behavior, 26(3), 235–240. https://doi-org.lib-e2.lib.ttu.edu/10.1002/(SICI)1098-2337(2000)26:3<235::AID-AB3>3.0.CO;2-L
Search.Two	128	Capio, C. M., Uiga, L., Lee, M. H., & Masters, R. S. W. (2020). Application of analogy learning in softball batting: Comparing novice and intermediate players. Sport, Exercise, and Performance Psychology, 9(3), 357–370. https://doi-org.lib-e2.lib.ttu.edu/10.1037/spy0000181
Search.Two	129	Gutiérrez, M., & Ruiz, L. M. (2009). Perceived motivational climate, sportsmanship, and students' attitudes toward physical educational classes and teachers. Perceptual and Motor Skills, 108(1), 308–326. https://doi-org.lib-e2.lib.ttu.edu/10.2466/PMS.108.1.308-326
Search.Two	130	Smith, R. E., Schutz, R. W., Smoll, F. L., & Ptacek, J. T. (1995). Development and validation of a multidimensional measure of sport-specific psychological skills: The Athletic Coping Skills Inventory-28. Journal of Sport & Exercise Psychology, 17(4), 379–398.
Search.Two	131	Pesce, C., Marchetti, R., Forte, R., Crova, C., Scatigna, M., Goudas, M., & Danish, S. J. (2016). Youth life skills training: Exploring outcomes and mediating mechanisms of a group-randomized trial in physical education. Sport,

		Exercise, and Performance Psychology, 5(3), 232–246. https://doi-org.lib-e2.lib.ttu.edu/10.1037/spy0000060
Search.Two	132	Johnson, M. B., Tenenbaum, G., Edmonds, W. A., & Castillo, Y. (2008). A comparison of the developmental experiences of elite and sub-elite swimmers: Similar developmental histories can lead to differences in performance level. Sport, Education and Society, 13(4), 453–475. https://doi-org.lib-e2.lib.ttu.edu/10.1080/13573320802445108
Search.Two	133	Martin, K. A., & Mack, D. (1996). Relationships between physical self-presentation and sport competition trait anxiety: A preliminary study. Journal of Sport & Exercise Psychology, 18(1), 75–82. https://doi-org.lib-e2.lib.ttu.edu/10.1123/jsep.18.1.75
Search.Two	134	Arruza, J. A., Telletxea, S., De Montes, L. G., Arribas, S., Balagué, G., Cecchini, J. A., & Brustad, R. J. (2009). Understanding the relationship between perceived development of the competition plan and sport performance: Mediating effects of self-efficacy and state depression. Perceptual and Motor Skills, 109(1), 304–314. https://doi-org.lib-e2.lib.ttu.edu/10.2466/pms.109.1.304-314
Search.Two	135	Besharat, M. A., & Pourbohlool, S. (2011). Moderating effects of self-confidence and sport self-efficacy on the relationship between competitive anxiety and sport performance. Psychology, 2(7), 760–765. https://doi-org.lib-e2.lib.ttu.edu/10.4236/psych.2011.27116
Search.Two	136	Miller, M., Carlyle, S., & Pease, R. (1992). The relationship between motivation and self-efficacy in competitive athletes participating in swimming, ice hockey, and basketball. Journal of Sport Behavior, 15(3), 201–208.
Search.Two	137	Vealey, R. S. (2019). A periodization approach to building confidence in athletes. Journal of Sport Psychology in Action, 10(1), 26–37. https://doi-org.lib-e2.lib.ttu.edu/10.1080/21520704.2018.1496213
Search.Two	138	Leonardi, T. J., Starapoli Martins, M. C., de Barros Gonçalves, C. E., Rodrigues Paes, R., & Gonçalves Moreira de Carvalho, H. J. (2019). Changes in tactical performance and self-efficacy on young female basketball players. / Variações na performance tática e na autoeficácia de jovens meninas atletas de basquetebol. Brazilian Journal of Kineanthropometry & Human Performance, 21, 1–9.
Search.Two	139	Nicholls, A. R., Polman, R., & Levy, A. R. (2010). Coping self-efficacy, pre-competitive anxiety, and subjective performance among athletes. European Journal of Sport Science, 10(2), 97–102. https://doi-org.lib-e2.lib.ttu.edu/10.1080/17461390903271592

Search.Two	140	Martin, J. J., & Gill, D. L. (1991). The Relationships Among Competitive Orientation, Sport-Confidence, Self-Efficacy, Anxiety, and Performance. Journal of Sport & Exercise Psychology, 13(2), 149–159.
Search.Two	141	Boat, R., & Taylor, I. M. (2015). Patterns of Change in Psychological Variables Leading up to Competition in Superior Versus Inferior Performers. Journal of Sport & Exercise Psychology, 37(3), 244–256.
Search.Two	142	Kuczka, K. K., & Treasure, D. C. (2005). Self-handicapping in competitive sport: Influence of the motivational climate, self-efficacy, and perceived importance. Psychology of Sport and Exercise, 6(5), 539–550. https://doi-org.lib-e2.lib.ttu.edu/10.1016/j.psychsport.2004.03.007
Search.Two	143	Kane, T. D., Marks, M. A., Zaccaro, S. J., & Blair, V. (1996). Self-efficacy, personal goals, and wrestlers' self-regulation. Journal of Sport & Exercise Psychology, 18(1), 36–48.
Search.Two	144	Bruton, A. M., Mellalieu, S. D., Shearer, D., Roderique-Davies, G., & Hall, R. (2013). Performance accomplishment information as predictors of self-efficacy as a function of skill level in amateur golf. Journal of Applied Sport Psychology, 25(2), 197–208. https://doi-org.lib-e2.lib.ttu.edu/10.1080/10413200.2012.705802
Search.Two	145	Smrdu, M. (2015). First-Person Experience of Optimal Sport Competition Performance of Elite Team Athletes. Kinesiology, 47(2), 169–178.
Search.Two	146	Vilar, L., Araújo, D., Davids, K., & Renshaw, I. (2012). The need for "representative task design" in evaluating efficacy of skills tests in sport: A comment on Russell, Benton and Kingsley (2010). Journal of Sports Sciences, 30(16), 1727–1730. https://doi-org.lib-e2.lib.ttu.edu/10.1080/02640414.2012.679674
Search.Two	147	Meijen, C., Jones, M., Mccarthy, P., Sheffield, D., & Allen, M. (2013). Cognitive and affective components of challenge and threat states. Journal of Sports Sciences, 31(8), 847–855.
Search.Two	148	Zabala, M., Morente-Sánchez, J., Mateo-March, M., & Sanabria, D. (2016). Relationship between self-reported doping behavior and psychosocial factors in adult amateur cyclists. The Sport Psychologist, 30(1), 68–75. https://doi-org.lib-e2.lib.ttu.edu/10.1123/tsp.2014-0168
Search.Two	149	Bueno, J., Capdevila, L., & Fernández-Castro, J. (2002). Sufrimiento competitivo y rendimiento en deportes de resistencia = Competitive suffering and performance in endurance sports. Revista de Psicología Del Deporte, 11(2), 209–226.
Search.Two	150	Middlemas, S., & Harwood, C. (2020). A pre-match video self-modeling intervention in elite youth football. Journal of

		Applied Sport Psychology, 32(5), 450–475. https://doi-org.lib-e2.lib.ttu.edu/10.1080/10413200.2019.1590481
Search.Two	151	Boes, R., Harung, H. S., Travis, F., & Pensgaard, A. M. (2014). Mental and physical attributes defining world-class Norwegian athletes: Content analysis of interviews. Scandinavian Journal of Medicine & Science in Sports, 24(2), 422–427. https://doi-org.lib-e2.lib.ttu.edu/10.1111/j.1600-0838.2012.01498.x
Search.Two	152	McAuley, E., & Gill, D. (1983). Reliability and validity of the physical self-efficacy scale in a competitive sport setting. Journal of Sport Psychology, 5(4), 410–418.
Search.Two	153	Weiss 1989 Head Over Heels With Success: The Relationship Between Self-Efficacy and Performance in Competitive Youth Gymnastics. (1989). Journal of Sport & Exercise Psychology, 11(4), 444–451.
Search.Two	154	Scanlan, T. K., & Passer, M. W. (1979). Factors Influencing the Competitive Performance Expectancies of Young Female Athletes. Journal of Sport Psychology, 1(3), 212–220.
Search.Two	155	Jones, M., Meijen, C., McCarthy, P. J., & Sheffield, D. (2009). A Theory of Challenge and Threat States in Athletes. International Review of Sport & Exercise Psychology, 2(2), 161–180.
Search.Two	156	Otten, M. (2009). Choking vs clutch performance: A study of sport performance under pressure. Journal of Sport & Exercise Psychology, 31(5), 583–601. https://doi-org.lib-e2.lib.ttu.edu/10.1123/jsep.31.5.583
Search.Two	157	Slimani, M., Chamari, K., Boudhiba, D., & Chéour, F. (2016). Mediator and moderator variables of imagery use-motor learning and sport performance relationships: a narrative review. Sport Sciences for Health, 12(1), 1–9.
Search.Two	158	Martin, J. J., & Gill, D. L. (1995). The relationships of competitive orientations and self-efficacy to goal importance, thoughts, and performance in high school distance runners. Journal of Applied Sport Psychology, 7(1), 50–62. https://doi-org.lib-e2.lib.ttu.edu/10.1080/10413209508406300
Search.Two	159	Lee, C. (1982). Self-efficacy as a Predictor of Performance in Competitive Gymnastics. Journal of Sport Psychology, 4(4), 405–409.
Search.Two	160	Hazell, J., Cotterill, S. T., & Hill, D. M. (2014). An exploration of pre-performance routines, self-efficacy, anxiety and performance in semi-professional soccer. European Journal of Sport Science, 14(6), 603–610. https://doi-org.lib-e2.lib.ttu.edu/10.1080/17461391.2014.888484

Search.Two	161	Duncan & McAuley 1987 Efficacy Expectations and Perceptions of Causality in Motor Performance. (1987). Journal of Sport Psychology, 9(4), 385–393.
Search.Two	162	Rojas, N. G. (2007). Implicaciones de la autoeficacia en el rendimiento deportivo = Implications of self-efficacy in sporting performance. Pensamiento Psicológico, 3(9), 21–32.
Search.Two	163	Fazlagić, A., & Belić, M. (2011). Lokus Kontrole I Flow Uspješnih Sportista. / Locus of Control and Flow of Successful Sportsman. Sportekspert, 4(1/2), 7–11.
Search.Two	164	Thelwell, R. C., & Maynard, I. W. (2000). Professional cricketers' perceptions of the importance of antecedents influencing repeatable good performance. Perceptual and Motor Skills, 90(2), 649–658. https://doi-org.lib-e2.lib.ttu.edu/10.2466/PMS.90.2.649-658
Search.Two	165	Miller, M. (1993). Efficacy strength and performance in competitive swimmers of different skill levels. International Journal of Sport Psychology, 24(3), 284–296.
Search.Two	166	Beaven, C. M. (2017). Mental Rehearsal Impacts Hormones and Subsequent Sport-Specific Performance. New Zealand Journal of Sports Medicine, 44(1), 41–42.
Search.Two	167	Hall, C. R., Munroe-Chandler, K. J., Fishburne, G. J., & Hall, N. D. (2009). The Sport Imagery Questionnaire for Children (SIQ-C). Measurement in Physical Education and Exercise Science, 13(2), 93–107. https://doi-org.lib-e2.lib.ttu.edu/10.1080/10913670902812713
Search.Two	168	Martin, J. J. (1995). Competitive orientation, self-efficacy and goal importance in Filipino marathoners. International Journal of Sport Psychology, 26(3), 348–358.
Search.Two	169	Bruton, A. M., Shearer, D. A., & Mellalieu, S. D. (2019). Who said "there is no 'I' in team"? The effects of observational learning content level on efficacy beliefs in groups. Psychology of Sport & Exercise, 45, N.PAG.
Search.Two	170	Toering, T. T., Elferink-Gemser, M. T., Jordet, G., & Visscher, C. (2009). Self-regulation and performance level of elite and non-elite youth soccer players. Journal of Sports Sciences, 27(14), 1509–1517. https://doi-org.lib-e2.lib.ttu.edu/10.1080/02640410903369919
Search.Two	171	Treasure, D. C., Monson, J., & Lox, C. L. (1996). Relationship between self-efficacy, wrestling performance, and affect prior to competition. The Sport Psychologist, 10(1), 73–83.
Search.Two	172	Chow, G. M., Hepler, T. J., & Feltz, D. L. (2007). The effects of group motivational processes on individual performance in women's volleyball. Journal of Sport & Exercise Psychology, 29, S152–S153.
Search.Two	173	Gernigon, C., Briki, W., & Eykens, K. (2010). The dynamics of psychological momentum in sport: The role of ongoing

		history of performance patterns. Journal of Sport & Exercise Psychology, 32(3), 377–400.
Search.Two	174	Hall, H. K., & Kerr, A. W. (1998). Predicting achievement anxiety: A social-cognitive perspective. Journal of Sport & Exercise Psychology, 20(1), 98–111.
Search.Two	175	Reifsteck, E. J., Anderson, S. N., Newton, J. D., & Maher, J. P. (2021). A practical guide and empirical example for implementing ecological momentary assessment in sport psychology research with athletes. Sport, Exercise, and Performance Psychology, 10(3), 408–422. https://doi-org.lib-e2.lib.ttu.edu/10.1037/spy0000252
Search.Two	176	Deeter, T. E. (1990). Re-modeling expectancy and value in physical activity. Journal of Sport & Exercise Psychology, 12(1), 86–91.
Search.Two	177	Psychountaki, M., & Zervas, Y. (2000). Competitive worries, sport confidence, and performance ratings for young swimmers. Perceptual and Motor Skills, 91(1), 87–94. https://doi-org.lib-e2.lib.ttu.edu/10.2466/PMS.91.5.87-94
Search.Two	178	Coutts, A. J., & Reaburn, P. (2008). Monitoring Changes in Rugby League Players' Perceived Stress and Recovery during Intensified Training. Perceptual & Motor Skills, 106(3), 904–916.
Search.Two	179	Kingston, K. M., & Hardy, L. (1997). Effects of different types of goals on processes that support performance. The Sport Psychologist, 11(3), 277–293.
Search.Two	180	Slimani, M., Tod, D., Chaabene, H., Miarka, B., & Chamari, K. (2016). Effects of Mental Imagery on Muscular Strength in Healthy and Patient Participants: A Systematic Review. Journal of Sports Science & Medicine, 15(3), 434–450.
Search.Two	181	Gil Martínez, J., Capafons Bonet, A., & Labrador Encinas, F. (1993). Variables físicas y psicológicas predictoras del rendimiento deportivo y del cambio terapeútico = Physical and psychological variables predictive of sports output and therapeutical change. Psicothema, 5(1), 97–110.
Search.Two	182	Turner, M. J., Jones, M. V., Sheffield, D., & Cross, S. L. (2012). Cardiovascular indices of challenge and threat states predict competitive performance. International Journal of Psychophysiology, 86(1), 48–57. https://doi-org.lib-e2.lib.ttu.edu/10.1016/j.ijpsycho.2012.08.004
Search.Two	183	Donovan, J. J., & Williams, K. J. (2003). Missing the mark: Effects of time and causal attributions on goal revision in response to goal-performance discrepancies. Journal of Applied Psychology, 88(3), 379–390. https://doi-org.lib-e2.lib.ttu.edu/10.1037/0021-9010.88.3.379
Search.Two	184	Wiggins, M. S. (1998). Anxiety intensity and direction: Preperformance temporal patterns and expectations in athletes. Journal of Applied Sport Psychology, 10(2), 201–

		211. https://doi-org.lib-e2.lib.ttu.edu/10.1080/10413209808406388
Search.Two	185	Suay, F., Salvador, A., González-Bono, E., Sanchís, C., Martínez, M., Martinéz-Sanchis, S., Simón, V. M., & Montoro, J. B. (1999). Effects of competition and its outcome on serum testosterone, cortisol and prolactin. Psychoneuroendocrinology, 24(5), 551–566. https://doi-org.lib-e2.lib.ttu.edu/10.1016/S0306-4530(99)00011-6
Search.Two	186	Greenlees, I. A., Nunn, R. L., Graydon, J. K., & Maynard, I. W. (1999). The relationship between collective efficacy and precompetitive affect in rugby players: Testing Bandura's model of collective efficacy. Perceptual and Motor Skills, 89(2), 431–440. https://doi-org.lib-e2.lib.ttu.edu/10.2466/PMS.89.6.431-440
Search.Two	187	Sheard, M., & Golby, J. (2006). Effect of a Psychological Skills Training Program on Swimming Performance and Positive Psychological Development. International Journal of Sport and Exercise Psychology, 4(2), 149–169. https://doi-org.lib-e2.lib.ttu.edu/10.1080/1612197X.2006.9671790
Search.Two	188	García-Naveira Vaamonde, A. (2018). Optimismo, autoeficacia general y competitividad en jóvenes atletas de alto rendimiento. / Optimism, general self-efficacy and competitiveness in young high-performance athletes. Cultura, Ciencia y Deporte, 13(37), 71–81.
Search.Two	189	García-Naveira Vaamonde, A. (2018). Autoeficacia y rendimiento en jugadores de fútbol = Self-efficacy and performance in soccer players. Cuadernos de Psicología Del Deporte, 18(2), 66–78.
Hand.searched.GoogleScholar	1	Rogowska AM, Tataruch R, Niedźwiecki K, Wojciechowska-Maszkowska B. The Mediating Role of Self-Efficacy in the Relationship between Approach Motivational System and Sports Success among Elite Speed Skating Athletes and Physical Education Students. International Journal of Environmental Research and Public Health. 2022; 19(5):2899. https://doi.org/10.3390/ijerph19052899
Hand.searched.GoogleScholar	2	Rafiee, S., Dana, A., Fallah, Z. (2022). Effect of Grit on Exercise Self-Efficacy and Participatory Performance in Elite Athletes. Sport Psychology Studies (ie, mutaleat ravanshenasi varzeshi), 11(39), 25-46. doi: 10.22089/spsyj.2019.6788.1731
Hand.searched.GoogleScholar	3	Brace AW, George K, Lovell GP (2020) Mental toughness and self-efficacy of elite ultra- marathon runners. PLoS ONE 15(11): e0241284.https://doi.org/10.1371/journal.pone.0241284

Hand.searched.GoogleScholar	4	Walter N, Nikoleizig L, Alfermann D. Effects of Self-Talk Training on Competitive Anxiety, Self-Efficacy, Volitional Skills, and Performance: An Intervention Study with Junior Sub-Elite Athletes. Sports. 2019; 7(6):148. https://doi.org/10.3390/sports7060148
Hand.searched.GoogleScholar	5	Karacam, A., & Adiguzel, N. S. (2019). Examining the relationship between referee performance and self-efficacy. European Journal of Educational Research, 8(1), 377-382. doi: 10.12973/eu-jer.8.1.377
Hand.searched.GoogleScholar	6	Samendinger, S., Hill, C. R., Hepler, T. J., & Feltz, D. L. (2019). Why Residuals Are Important in the Self-Efficacy–Performance Relationship Analysis: A Study Across 12 Cycling Sessions. Journal of Physical Activity & Health, 16(6), 455–460.
Hand.searched.GoogleScholar	7	Sari 2015. An Investigation of Imagery, Intrinsic Motivation, Self-efficacy and Performance in Athletes
Hand.searched.GoogleScholar	8	Sari 2015. Does goal orientation...
Hand.searched.GoogleScholar	9	Fitzimmons, P. A., Landers, D. M., Thomas, J. R., & van der Mars, H. (1991). Does self-efficacy predict performance in experienced weightlifters? / La competence percue predit-elle la performance chez des halterophiles experimentes. Research Quarterly for Exercise & Sport, 62(4), 424–431.
Hand.searched.GoogleScholar	10	Miller, M., Carlyle, S., & Pease, R. (1992). The relationship between motivation and self-efficacy in competitive athletes participating in swimming, ice hockey, and basketball. Journal of Sport Behavior, 15(3), 201–208.
Hand.searched.GoogleScholar	11	Stajkovic, A. D., & Luthans, F. (1998). Self-efficacy and work-related performance: A meta-analysis. Psychological Bulletin, 124(2), 240. https://doi-org.lib-e2.lib.ttu.edu/10.1037/0033-2909.124.2.240
Hand.searched.GoogleScholar	12	Bray, S. R., Balaguer, I., & Duda, J. L. (2004). The relationship of task self-efficacy and role efficacy beliefs to role performance in Spanish youth soccer. Journal of Sports Sciences, 22(5), 429–437.
Hand.searched.GoogleScholar	13	Maeda et al. 2003
Hand.searched.GoogleScholar	14	Singh, T., Bhardwaj, G., & Bhardwaj, V. (2009). Effect of self-efficacy on the performance of athletes. Journal of Exercise Science and Physiotherapy, 5(2), 110–114. https://search.informit.org/doi/10.3316/informit.856176212483236
Hand.searched.GoogleScholar	15	Barker, J., Jones, M., & Greenlees, I. (2010). Assessing the Immediate and Maintained Effects of Hypnosis on Self-Efficacy and Soccer Wall-Volley Performance. Journal of Sport & Exercise Psychology, 32(2), 243–252.

Hand.searched.GoogleScholar	16	Llewellyn, D. J., Sanchez, X., Asghar, A., & Jones, G. (2008). Self-efficacy, risk taking and performance in rock climbing. Personality and Individual Differences, 45(1), 75–81. https://doi-org.lib-e2.lib.ttu.edu/10.1016/j.paid.2008.03.001
Hand.searched.GoogleScholar	17	Ortega, E., Olmedilla, A., de Baranda, P. S., & Gómez, M. Á. (2009). Relationship between the level of self-efficacy, performance indicators, and participation in youth basketball. Revista de Psicología Del Deporte, 18(Suppl), 337–342.
Hand.searched.GoogleScholar	18	Cumming, J., Nordin, S. M., Horton, R., & Reynolds, S. (2006). Examining the Direction of Imagery and Self-Talk on Dart-Throwing Performance and Self Efficacy. The Sport Psychologist, 20(3), 257–274.
Hand.searched.GoogleScholar	19	Hayslip Jr., B., Raab, C. M., Baczewski, P. C., & Petrie, T. A. (2010). The Development and Validation of the Golf Self-Efficacy Scale. Journal of Sport Behavior, 33(4), 427–441.
Hand.searched.GoogleScholar	20	Chang, Y. K., Ho, L. A., Lu, F. J. H., Ou, C. C., Song, T. F., & Gill, D. (2014). Self-talk and softball performance: The role of self-talk nature, motor task characteristics, and self-efficacy in novice softball players. Psychology of Sport and Exercise, 15(1), 139-145.
Hand.searched.GoogleScholar	21	Pete Coffee, Tim Rees & S. Alexander Haslam (2009) Bouncing back from failure: The interactive impact of perceived controllability and stability on self-efficacy beliefs and future task performance, Journal of Sports Sciences, 27:11, 1117-1124, DOI: 10.1080/02640410903030297
Hand.searched.GoogleScholar	22	Lisa J. van Raalte & Karlee A. Posteher (2019) Examining social support, self-efficacy, stress, and performance, in U.S. Division I collegiate student-athletes' academic and athletic lives, Journal for the Study of Sports and Athletes in Education, 13:2, 75-96, DOI: 10.1080/19357397.2019.1635419
Hand.searched.GoogleScholar	23	Pullen, B.J.; Oliver, J.L.; Lloyd, R.S.; Knight, C.J. Relationships between Athletic Motor Skill Competencies and Maturity, Sex, Physical Performance, and Psychological Constructs in Boys and Girls. Children 2022, 9, 375. https://doi.org/10.3390/children9030376
Search of 2022-23		
Hand-searching SPORTDiscus	Include	Aikawa, S., & Takai, H. (2021). Relationship Between Imagery Ability, Performance, and Variables Related to Performance. The Sport Psychologist, 35(2), 123–130. https://doi.org/10.1123/tsp.2019-0165
Review of Jekauc efficacy studies	Include	Beauchamp, M. R., & Whinton, L. C. (2005). Self-Efficacy and Other-Efficacy in Dyadic Performance: Riding as One in Equestrian Eventing. Journal of Sport and Exercise Psychology, 27(2), 245–252. https://doi.org/10.1123/jsep.27.2.245

EBSCO Search 1	No competition	Schüler, J., Stähler, J., & Wolff, W. (2023). Mind-over-body beliefs in sport and exercise: A driving force for training volume and performance, but with risks for exercise addiction. Psychology of Sport & Exercise, 68, N.PAG.
	Review, no original data	Williamson, O., Swann, C., Bennett, K. J. M., Bird, M. D., Goddard, S. G., Schweickle, M. J., & Jackman, P. C. (2022). The performance and psychological effects of goal setting in sport: A systematic review and meta-analysis. International Review of Sport and Exercise Psychology. https://doi-org.lib-e2.lib.ttu.edu/10.1080/1750984X.2022.2116723
EBSCO Search 2	No competition	Ortiz Romero, M. T., Garrido Guzmán, M. E., & Castañeda Vázquez, C. (2022). Autoeficacia y resiliencia: diferencias entre deportistas practicantes de fitness/culturismo y no deportistas. / Self-efficacy and resilience: differences between fitness/bodybuilding athletes and nonathletes. Retos: Nuevas Perspectivas de Educación Física, Deporte y Recreación, 44, 232–241.
	No competition	Bastik, C., & Gümüsdag, H. (2022). A Comparison of Mental Training Skills Levels of Handball and Basketball Players. International Journal of Curriculum and Instruction, 14(3), 2391–2399.
	Not peer reviewed	Troutman, T. D. (2023). A qualitative exploration: Factors impacting the academic achievement of high school student-athletes [ProQuest Information & Learning]. In Dissertation Abstracts International Section A: Humanities and Social Sciences (Vol. 84, Issue 5–A).
	no performance measure	Meijen, C., McCormick, A., Anstiss, P. A., & Marcora, S. M. (2022). "Short and Sweet": A Randomized Controlled Initial Investigation of Brief Online Psychological Interventions With Endurance Athletes. Sport Psychologist, 36(1), 20–28.
	no self-efficacy measure	Duncan, M. J., Eyre, E. L. J., Noon, M. R., Morris, R., Thake, C. D., Clarke, N. D., & Cunningham, A. J. (2022). Actual and perceived motor competence mediate the relationship between physical fitness and technical skill performance in young soccer players. European Journal of Sport Science, 22(8), 1196–1203.
	No competition	Schneider, J., Matheson, E. L., Tinoco, A., Gentili, C., White, P., Boucher, C., Silva-Breen, H., Goorevich, A., Diedrichs, P. C., & LaVoi, N. M. (2023). Body confident coaching: A pilot randomized controlled trial evaluating the acceptability of a web-based body image intervention for coaches of adolescent girls. Journal of Applied Sport Psychology. https://doi-org.lib-e2.lib.ttu.edu/10.1080/10413200.2023.2212023
	Review, no original data	Hebard, S. P., Bissett, J. E., Kroshus, E., Beamon, E. R., & Reich, A. (2023). A Content Analysis of Mental Health

		Literacy Education for Sport Coaches. Journal of Clinical Sport Psychology, 17(2), 165–175.
	Repeat from Search 1	Williamson, O., Swann, C., Bennett, K. J. M., Bird, M. D., Goddard, S. G., Schweickle, M. J., & Jackman, P. C. (2022). The performance and psychological effects of goal setting in sport: A systematic review and meta-analysis. International Review of Sport and Exercise Psychology. https://doi-org.lib-e2.lib.ttu.edu/10.1080/1750984X.2022.2116723
Google scholar 2023 August 30 search	no methodology section, no indication of actual competition	Yang, X., & Mojtahe, K. (2023). Understanding the Role of Self-Efficacy in Sports Performance: A Longitudinal Study. Revista de Psicología del Deporte (Journal of Sport Psychology), 32(1), 311-319.
Reviewed page 1 of results	No self-efficacy (CSAI-2 confidence)	Mercader-Rubio, I., Gutiérrez Ángel, N., Silva, S., Moisão, A., & Brito-Costa, S. (2023). Relationships between somatic anxiety, cognitive anxiety, self-efficacy, and emotional intelligence levels in university physical education students. Frontiers in Psychology, 13, 1059432.
	No competition	Volgemute, K., Vazne, Z., Krauksta, D., & Cosma, G. A. (2023). The improvement of athletes' imagery ability and physical self-efficacy for the growth of athletic achievements in sport model. Journal of Physical Education and Sport, 23(3), 748-755.
	cross-sectional, no actual competition	Reverdito, R. S., Fonseca, S., Lopes, A., Aires, K., Santos Alves, L., Alves de Lima, L., ... & Gonçalves, C. (2023). Sources of Sport Satisfaction and Perceived Self-Efficacy Among Youth in a Competitive Environment. Perceptual and Motor Skills, 00315125231167460.
	no actual competition, retrospective coach/parent ratings of athletes	Di Corrado, D., Sagone, E., Buscemi, A., & Coco, M. (2023). The Relationship between Anger Expression and Performance Score in Parents and Coaches: The Mediating Role of Self-Efficacy and Assertiveness. International Journal of Environmental Research and Public Health, 20(7), 5372.
	Not with athletes	Richlan, F., Thürmer, J. L., Braid, J., Kastner, P., & Leitner, M. C. (2023). Subjective experience, self-efficacy, and motivation of professional football referees during the COVID-19 pandemic. Humanities and Social Sciences Communications, 10(1), 1-15.
	Pre-print, not peer reviewed	Yu, C., Kao, C., Wu, J., Hung, E., Chang, W., Yang, R., ... & Hung, T. (2023). Effects of self-efficacy on frontal midline theta power and golf putting performance.
	Not sport skill	Zulkipli, U. N., Nor, M. A. M., Lubis, J., & Shaari, J. S. (2023, June). Relationship between Self-Efficacy and Push-Up Performance among Male Gymnasium Members.

		In Proceedings of the 8th ACPES (ASEAN Council of Physical Education and Sport) International Conference, ACPES 2022, October 28th–30th, 2022, Medan, North Sumatera, Indonesia.
	Not sport skill	Cudicio, A., & Agosti, V. (2023). THE LINK BETWEEN PHYSICAL PERFORMANCE AND SELF-EFFICACY: HOW MUCH CAN I JUMP?. ITALIAN JOURNAL OF HEALTH EDUCATION, SPORT AND INCLUSIVE DIDACTICS, 7(2).
Google scholar 2022 August 30 search	study 1: cross-sectional, not prior to performance, hypothetical competition; study 2: no performance	Kwon, T., Shin, S., & Shin, M. (2022). The effect of observational learning on self-efficacy by sport competition condition, performance level of team members, and whether you win or lose. International Journal of Environmental Research and Public Health, 19(16), 10148.
Reviewed page 1 of results	Not sport skill	Horcajo, J., Santos, D., & Higuero, G. (2022). The effects of self-efficacy on physical and cognitive performance: An analysis of meta-certainty. Psychology of Sport and Exercise, 58, 102063.
	cross-sectional, not prior to performance	Wang, C. M., Hong, J. C., Ye, J. H., & Ye, J. N. (2022). The relationship among gameplay self-efficacy, competition anxiety, and the performance of eSports players. Entertainment Computing, 42, 100489.
	No actual competition	Rogowska, A. M., Tataruch, R., Niedźwiecki, K., & Wojciechowska-Maszkowska, B. (2022). The mediating role of self-efficacy in the relationship between approach motivational system and sports success among elite speed skating athletes and physical education students. International journal of environmental research and public health, 19(5), 2899.
	No actual competition	Stephen, S. A., Habeeb, C. M., & Arthur, C. A. (2022). Congruence of efficacy beliefs on the coach-athlete relationship and athlete anxiety: Athlete self-efficacy and coach estimation of athlete self-efficacy. Psychology of sport and exercise, 58, 102062.
	No competition	Satjawathee, T., Ma, S. C., Shu, S. T., & Chang, C. H. (2022). The moderating effect of self-efficacy on fitness use innovativeness and usage pattern. Sustainability, 15(1), 586.
	Not athletes	Golsanamlou, M., Hamidi, M., Rajabi Noush Abadi, H., & Sajjadi, S. N. (2022). The Effect of Empowerment on Physical Education Teachers' Self-efficacy and Job Performance. Research on Educational Sport, 10(26), 220-197.
	Not athletes	Baniasadi, T., Ranjbari, S., Mofrad, S. K., & Amir, D. (2022). Associations between device-measured physical activity

		and balance performance in children: Mediating role of motor self-efficacy. Biomedical Human Kinetics, 14(1), 252-258.
	No competition	Oguntuase, S. B., & Sun, Y. (2022). Effects of mindfulness training and locus of control on self-efficacy of professional football players in Nigeria. Journal of Physical Education & Sport, 22(5).
	No competition	Piussi, R., Beischer, S., Thomeé, R., Thomeé, C., Sansone, M., Samuelsson, K., & Senorski, E. H. (2022). Greater psychological readiness to return to sport, as well as greater present and future knee-related self-efficacy, can increase the risk for an anterior cruciate ligament re-rupture: a matched cohort study. Arthroscopy: The Journal of Arthroscopic & Related Surgery, 38(4), 1267-1276.
	No competition	Stanković, N., Todorović, D., Milošević, N., Mitrović, M., & Stojiljković, N. (2022). Aggressiveness in judokas and team athletes: Predictive value of personality traits, emotional intelligence and self-efficacy. Frontiers in psychology, 12, 824123.
	No competition	Liu, C. (2022). Research on the influence of college students' participation in sports activities on their sense of inferiority based on self-esteem and general self-efficacy. Frontiers in Psychology, 13, 994209.
	cross-sectional, not prior to performance, and no description of sport performance (how measured, etc.)	Ahmad, N., Marwat, M. K., Iqbal, Y., Nizami, R., Shah, M., Saman, S., & Mehmood, K. (2022). IMPACT OF SPORT-PERFECTIONISM AND SELF-EFFICACY ON ACHIEVEMENT MOTIVATION AND SPORTS PERFORMANCE (A CASE STUDY OF INDIVIDUAL DIFFERENCES OF UNIVERSITY STUDENTS OF DISTRICT LAHORE). PalArch's Journal of Archaeology of Egypt/Egyptology, 19(2), 1275-1285.
	no data with efficacy and shot performance	Metan, H., & Küçük, V. (2022). The Effect of Psychological Skill Training Program and Positive Feedback on Handball Player's Self-Efficacy Beliefs and their Shot Accuracy. Annals of Applied Sport Science, 10(3), 0-0.
